# Cellular IAP proteins and LUBAC differentially regulate necrosome-associated RIP1 ubiquitination

**DOI:** 10.1038/cddis.2015.158

**Published:** 2015-06-25

**Authors:** M C de Almagro, T Goncharov, K Newton, D Vucic

**Affiliations:** 1 Department of Early Discovery Biochemistry, Genentech Inc., South San Francisco, CA 94080, USA; 2 Department of Physiological Chemistry, Genentech Inc., South San Francisco, CA 94080, USA

## Abstract

Necroptosis is a caspase-independent regulated type of cell death that relies on receptor-interacting protein kinases RIP1 (receptor-interacting protein kinases 1) and RIP3. Tumor necrosis factor-*α* (TNF*α*)-stimulated assembly of the TNFR1 (TNF receptor 1)-associated signaling complex leads to the recruitment of RIP1, whose ubiquitination is mediated by the cellular inhibitors of apoptosis (c-IAPs). Translocation of RIP1 to the cytoplasm and association of RIP1 with the necrosome is believed to correlate with deubiquitination of RIP1. However, we found that RIP1 is ubiquitinated with K63 and linear polyubiquitin chains during TNF*α*, IAP antagonist BV6 and caspase inhibitor zVAD-fmk-induced necroptotic signaling. Furthermore, ubiquitinated RIP1 is associated with the necrosome, and RIP1 ubiquitination in the necrosome coincides with RIP3 phosphorylation. Both cellular IAPs and LUBAC (linear ubiquitin chain assembly complex) modulate RIP1 ubiquitination in IAP antagonist-treated necrotic cells, but they use different mechanisms. c-IAP1 regulates RIP1 recruitment to the necrosome without directly affecting RIP1 ubiquitination, whereas HOIP and HOIL1 mediate linear ubiquitination of RIP1 in the necrosome, but are not essential for necrosome formation. Knockdown of the E3 ligase c-IAP1 decreased RIP1 ubiquitination, necrosome assembly and necroptosis induced by TNF*α*, BV6 and zVAD-fmk. c-IAP1 deficiency likely decreases necroptotic cell death through the activation of the noncanonical NF-*κ*B pathway and consequent c-IAP2 upregulation. The ability to upregulate c-IAP2 could determine whether c-IAP1 absence will have a positive or negative impact on TNF*α*-induced necroptotic cell death and necrosome formation. Collectively, these results reveal unexpected complexity of the roles of IAP proteins, IAP antagonists and LUBAC in the regulation of necrosome assembly.

Proper cell death regulation is critical for tissue homeostasis with impaired or excessive cell death contributing to numerous pathologies.^1, 2^ The best understood form of regulated cell death is apoptosis, which activates cysteine proteases called caspases.^3^ Recent advances have defined another type of cell death called necroptosis, a highly regulated process that occurs when caspases are inhibited. Necroptosis involves activation of receptor-interacting protein kinases 1 (RIP1, aka RIPK1) and RIP3 and has distinct cellular features, which include early loss of plasma membrane integrity, organelle swelling and inflammation.^4, 5, 6^

Necroptosis can be induced by TNF*α* (tumor necrosis factor *α*), TLRs (toll-like receptors) or by viral infection.^2^ Binding of TNF*α* to TNFR1 (TNF receptor 1) triggers the recruitment of adaptor proteins TRADD and TRAF2, ubiquitin E3 ligases cellular inhibitor of apoptosis (c-IAP) proteins and RIP1 to form the receptor-associated signaling complex-I.^7^ Within complex-I, c-IAPs promote ubiquitination of RIP1 with K11- and K63-linked polyubiquitin chains.^8, 9, 10, 11^ RIP1 ubiquitination serves as a platform for the recruitment of other components of the NF-*κ*B and mitogen-activated protein kinase signaling pathways including LUBAC (linear ubiquitin chain assembly complex).^12, 13^ LUBAC is an ubiquitin E3 ligase complex that attaches linear polyubiquitin chains on RIP1 and consists of the catalytic subunit HOIP and the auxiliary proteins HOIL1 and sharpin.^14, 15, 16, 17^ Diverse ubiquitination chains assembled on RIP1 are proposed to keep RIP1 within complex-I.^10, 13^

c-IAP deficiency, which can be achieved by gene targeting or by using IAP antagonists that stimulate c-IAP proteasomal degradation, permits non-ubiquitinated RIP1 to leave the TNFR1-associated complex and join FADD, caspase-8/10 and FLIP in cytoplasmic complex-II.^10, 18, 19, 20, 21^ If caspase activation is inhibited, RIP1 engages RIP3 to form the necrosome signaling complex and switches cell death from apoptosis to necroptosis.^22^ The kinase activities of RIP1 and RIP3 are essential for necroptosis and the RIP1 kinase inhibitor, necrostatin-1, blocks necroptotic cell death.^23, 24, 25^ Following autophosphorylation of RIP1 and RIP3, phosphorylated RIP3 recruits its substrate, the pseudokinase MLKL (mixed lineage kinase domain-like). Phosphorylated MLKL oligomerizes and translocates to membranes resulting in cell rupture.^26, 27, 28, 29, 30^

RIP1 is a key factor in determining both whether a cell lives or dies, and the mode of death.^31^ Ubiquitination of RIP1 is essential for survival signaling,^32^ but it may also be integral to necroptosis. In this study, we found that necrosome-associated RIP1 was ubiquitinated, at least in part, by linear polyubiquitin chains assembled by HOIP and HOIL1. Interestingly, this linear ubiquitination on RIP1 was dispensable for necroptosis. In contrast, c-IAPs were dispensable for ubiquitination of RIP1 within the necrosome. Loss of c-IAP1 resulted in the upregulation of c-IAP2 and noncanonical NF-*κ*B signaling in some cells with a concomitant decrease in TNF*α* plus IAP antagonist-induced necrosome formation and necroptosis. These results reveal unexpected complexity in the roles of c-IAP1 and c-IAP2 in regulating necrosome assembly.

## Results

### RIP1 is ubiquitinated in the necrosome

**Figure 1:**
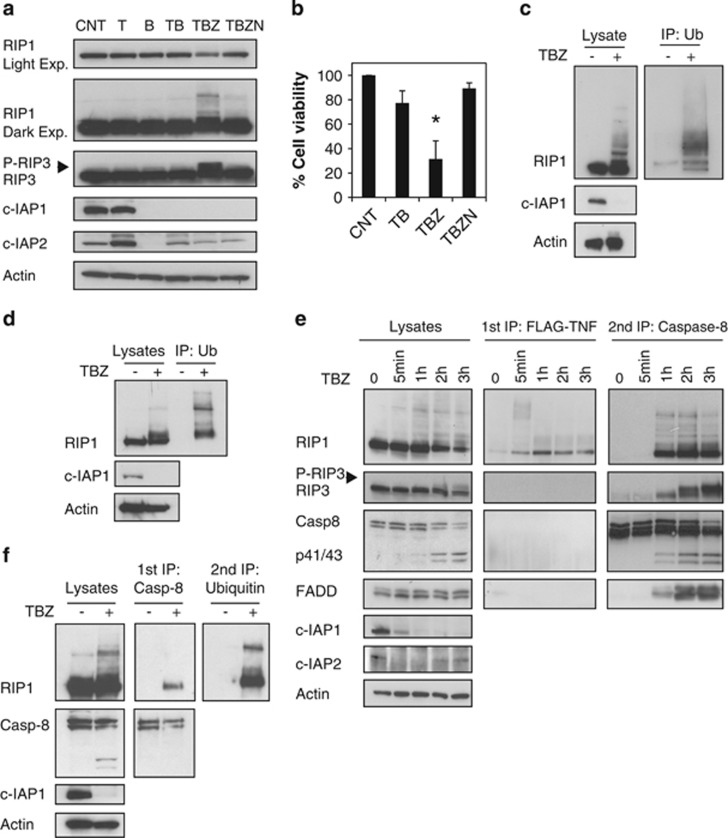
Rip1 is ubiquitinated during necroptotic signaling. (**a**) HT29 cells were treated for 3 h with TNF*α* 20 ng/ml (T), BV6 2 *μ*M (B), zVAD 20 *μ*M (Z) and Nec-1 30 *μ*M (N) as indicated. Cell lysates were analyzed by western blotting with the indicated antibodies. (**b**) HT29 cells were treated for 20 h with TNF*α* 20 ng/ml (T), BV6 0.5 *μ*M (B), zVAD 20 *μ*M (Z) and Nec-1 30 *μ*M (N). Cell viability was assessed by CellTiter-Glo. Data are mean±S.E.M. values of at least three experiments (*P<0.05). (**c**) HT29 cells were treated for 2 h with TNF*α* 20 ng/ml (T), BV6 2 *μ*M (B) and zVAD 20 *μ*M (Z). Denatured cell lysates were immunoprecipitated with ubiquitin antibody and analyzed by western blotting. (**d**) MEF cells were treated for 2 h with TNF*α* 100 ng/ml (T), BV6 2 *μ*M (B) and zVAD 20 *μ*M (Z). Denatured cell lysates were immunoprecipitated with ubiquitin antibody and analyzed by western blotting with the indicated antibodies. (**e**) HT29 cells were treated for the indicated periods of time with Flag-TNF*α* 1 *μ*g/ml (T), BV6 2 *μ*M (B) and zVAD 20 *μ*M (Z). Cell lysates were first immunoprecipitated with Flag beads, and the supernatants underwent a second immunoprecipitation with caspase-8 antibody. The pull-downs and lysates were analyzed by western blotting with the indicated antibodies. (**f**) HT29 cells were treated for 2 h with TNF*α* 20 ng/ml (T), BV6 2 *μ*M (B) and zVAD 20 *μ*M (Z). Following the first immunoprecipitation with caspase-8 antibody, caspase-8-associated complex was disrupted and supernatants underwent a second immunoprecipitation with ubiquitin antibody. The pull-downs and lysates were analyzed by western blotting with the indicated antibodies

RIP1 is ubiquitinated within the TNFR1-associated signaling complex-I, and RIP1 deubiquitination is reported to be necessary for the assembly of cytoplasmic complex-II.^10, 33, 34^ To investigate the ubiquitination status of RIP1 during necroptosis, human colon carcinoma HT29 cells were induced to undergo necroptosis with TNF*α*, IAP antagonist BV6 and the pancaspase inhibitor zVAD-fmk (combination hereafter referred as TBZ). Surprisingly, immunoblotting of total RIP1 revealed slower migrating forms of RIP1 at 3 h after TBZ but not after the apoptotic stimulus of TNF*α* and BV6 or the individual stimuli (Figure 1a and Supplementary Figure S1A). This modified form of RIP1 coincided with a slower migrating form of RIP3 (Figure 1a), which was sensitive to phosphatase treatment and therefore represented phosphorylated RIP3 (Supplementary Figure S1B). Phosphorylated RIP3 and what appeared to be ubiquitinated RIP1 in cells treated with TBZ was markedly reduced by the RIP1 kinase inhibitor necrostatin-1 (Nec-1) (Figure 1a). Consistent with previous reports,^26^ Nec-1 protected HT29 cells from killing by TBZ (Figure 1b). Similar modification of RIP1 was observed in another cell line commonly used to study necroptosis, the mouse cell line L929 (Supplementary Figure S1C).

We confirmed that RIP1 was indeed ubiquitinated during necroptosis by treating HT29 and mouse embryonic fibroblasts (MEFs) with TNF*α*+BV6+zVAD and immunoblotting for RIP1 after immunoprecipitating ubiquitinated proteins (Figures 1c and d). To determine if RIP1 within the necrosome was ubiquitinated, we treated HT29 cells with Flag-tagged TNF, BV6 and zVAD-fmk and then isolated TNFR1-associated complex-I (Figure 1e, middle panels). Antibodies to caspase-8 were then used to capture the necrosome/complex-II from complex-I-depleted lysates (Figure 1e, right panels). Following early ubiquitination of TNFR1-associated RIP1 (5 min after stimulation), RIP1 ubiquitination decreases and RIP1 leaves the TNFR1 complex to bind caspase-8 where it is ubiquitinated again (Figure 1e). Similar TBZ-induced and caspase-8-associated RIP1 ubiquitination was observed in several additional cell lines (Supplementary Figure S1D). To confirm that the modifications observed in caspase-8-bound RIP1 upon TBZ treatment were ubiquitination, a first immunoprecipitation with caspase-8 was performed, followed by disruption of the complex and a second immunoprecipitation with ubiquitin antibody (Figure 1f). Collectively, these data demonstrate that ubiquitinated RIP1 is part of the necrosome complex together with caspase-8, FADD and phosphorylated RIP3.

### Necroptosis stimulates K63 and linear chain-linked polyubiquitination of RIP1

**Figure 2:**
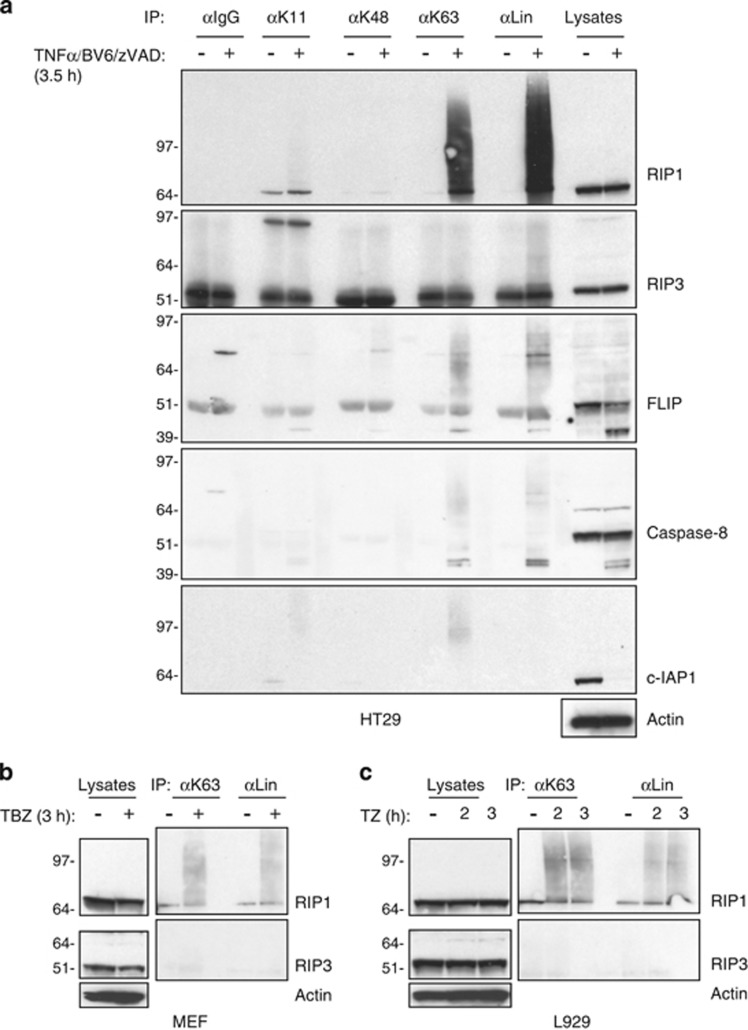
RIP1 undergoes K63 and linear chain-linked polyubiquitination during necroptosis. (**a**) HT29 cells were treated with TNF*α* 20 ng/ml (T), BV6 2 *μ*M (B) and zVAD (20 *μ*M) for 3.5 hours. Cells were lysed in 6 M urea buffer and immunoprecipitated using linkage-specific anti-ubiquitin antibodies or control antibody. Immunoprecipitated proteins were detected using indicated antibodies. (**b** and **c**) MEF (**b**) or L929 (**c**) cells were not treated or treated with TNF*α* (100 ng/ml), BV6 (2 *μ*M) and zVAD (20 *μ*M) for 3 h (**b**) or with TNF*α* (10 ng/ml) and zVAD (20 *μ*M) for 2 or 3 h (**c**). Cells were lysed and immunoprecipitated as in (**a**) and immunoprecipitated proteins were detected using indicated antibodies

To investigate the nature of necroptotic ubiquitination, we used ubiquitin chain-specific antibodies to determine the ubiquitination status of several proteins associated with necroptotic signaling (Figure 2 and Supplementary Figures S2 and 3). We treated HT29 cells with TBZ to induce necroptosis, lysed them in denaturing urea buffer and immunoprecipitated with ubiquitin chain-specific antibodies. Our experiment revealed strong, necroptosis stimulus-dependent RIP1 ubiquitination with K63 and linear polyubiquitin chains (Figure 2a). We also observed modest modification of FLIP and caspase-8 (Figure 2a). In addition, we also noticed marginal linear ubiquitination of RIP3, and K63-linked ubiquitination of c-IAP1 protein that was not completely degraded (Figure 2a). In addition to HT29 cells, TBZ-induced necroptosis stimulated K63-linked and linear RIP1 polyubiquitination in other human cell lines as well (Supplementary Figure S2A). To verify if this ubiquitination pattern is selective to necroptotic stimulus, we treated HT29 cells with TNF and BV6 to activate apoptosis (Supplementary Figure S2B). TB produced minimal RIP1 ubiquitination at 3.5 h when compared with TBZ (Figure 2a and Supplementary Figures S2A and B).

Next, we examined if this pattern of necroptotic ubiquitination could be observed in murine cells. To that end, we treated MEFs with TBZ and L929 cells with TZ and investigated ubiquitination using ubiquitin chain-specific antibodies. Again, we observed significant K63 and linear polyubiquitination of RIP1 but not of RIP3 or caspase-8 (Figures 2b and c and Supplementary Figure S2C). We further expanded our analyses of necroptotic ubiquitination by investigating LPS-induced cell death in L929 cells (Supplementary Figure S3A). Treatment of L929 cells with LPS, BV6 and zVAD (LBZ) prompted K63-linked and linear RIP1 ubiquitination, but not as prominently as TZ treatment (Supplementary Figures S3B and C). On the other hand, LPS alone or together with zVAD did not stimulate RIP1 ubiquitination, although IRAK1, a critical component of LPS-induced signaling, was modified by K63 and linear ubiquitin linkages (Supplementary Figures S3B and C). Taken together, these results indicate that RIP1 is the primary target of necroptotic ubiquitination, predominantly with K63 and linear chain linkages.

### c-IAPs do not mediate ubiquitination of RIP1 during necroptosis

**Figure 3:**
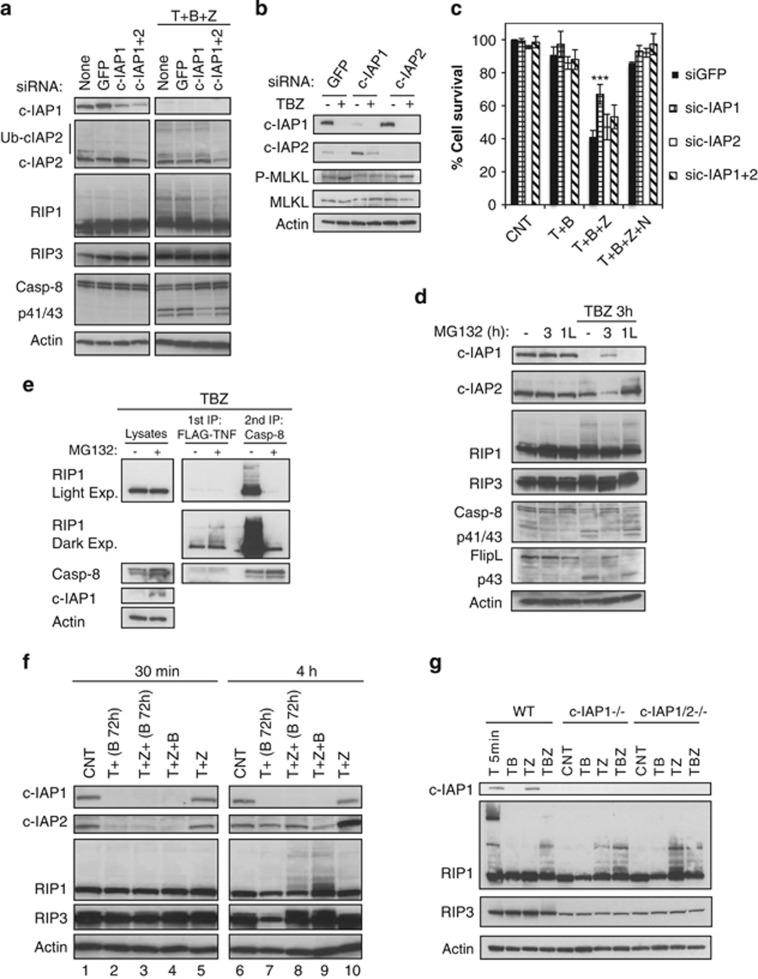
c-IAPs do not mediate RIP1 ubiquitination in necroptotic cell death. (**a**–**c**) HT29 cells were transfected with the indicated small interfering RNAs (siRNAs) for 72 h. (**a**) After 72 h, cells were left untreated or treated with TNF*α* 20 ng/ml (T), BV6 0.5 *μ*M (B), zVAD 20 *μ*M (Z) for 3 h and lysates were analyzed by western blotting with the indicated antibodies. (**b**) HT29 cells were left untreated or treated with TNF*α* 20 ng/ml (T), BV6 2 *μ*M (B) and zVAD 20 *μ*M (Z) for 2 h. Lysates were analyzed by western blotting with the indicated antibodies. (**c**) HT29 cells were treated with TNF*α* 20 ng/ml (T), BV6 0.5 *μ*M (B), zVAD 20 *μ*M (Z) and Nec-1 30 *μ*M (N) for 20 h and viability was assessed by CellTiter-Glo. Data are mean±S.E.M. values of at least four experiments (***P<0.005). (**d**) HT29 cells were left untreated or treated for 3 h with TNF*α* 20 ng/ml (T), BV6 2 *μ*M (B) and zVAD 20 *μ*M (Z). MG132 20 *μ*M was not applied (−), or was administered 30 min before TBZ treatment in 3 h cells, or applied for the last hour of TBZ treatment in 1 L cells. Cell lysates were analyzed as in (**a**). (**e**) HT29 cells were treated with Flag-TNF*α* 1 *μ*g/ml (T), BV6 2 *μ*M (B) and zVAD 20 *μ*M (Z) for 3 h and MG132 (20 *μ*M) as indicated 30 min before TBZ treatment. Cell lysates were first immunoprecipitated with Flag beads, and the supernatants underwent a second immunoprecipitation with caspase-8 antibody. The pull-downs and lysates were analyzed by western blotting with the indicated antibodies. (**f**) HT29 cells were left untreated or treated for 72 h with BV6 0.5 *μ*M (B, 72 h). After 72 h, cells were treated for the indicated times with TNF*α* 20 ng/ml (T), BV6 0.5 *μ*M (B) and zVAD 20 *μ*M (Z). (**g**) WT, c-IAP1^−/−^ KO and c-IAP1/2^−/−^ DKO MEFs were treated with TNF*α* 100 ng/ml (T), BV6 2 *μ*M (B) and/or zVAD 20 *μ*M (Z) for 3 h, except for the sample in the first lane (5 min with TNF*α*). (**f** and **g**) Cell lysates were analyzed by western blotting with the indicated antibodies

Given that c-IAP proteins are E3 ligases for RIP1,^10, 11^ we investigated their role in RIP1 ubiquitination during necroptosis. Knockdown of c-IAP1 caused a noticeable reduction in RIP1 ubiquitination at 3 h after TBZ (Figure 3a). c-IAP1 downregulation also reduced phosphorylation of MLKL and improved cell viability in response to TBZ (Figures 3b and c and Supplementary Figures S4A and B). c-IAP2 knockdown did not affect cell viability in the same way as c-IAP1 knockdown (Figures 3b and c). However, double knockdown of c-IAP1 and c-IAP2 restored necroptosis levels (Figure 3c and Supplementary Figure S4B).

To assess if c-IAP1 was the E3 ligase for RIP1 during necroptosis, cells were pretreated with the proteasome inhibitor MG132 to limit proteasomal degradation of c-IAP1 triggered by BV6. The expectation was that we would see more RIP1 ubiquitination. Contrary to expectations, pretreatment of cells (3 h time point) with MG132 decreased RIP1 ubiquitination, although c-IAP1 was (at least partly) stabilized (Figure 3d). This was accompanied by the stabilization of procaspase-8 and FLIP, and a decrease in RIP3 phosphorylation. Administration of MG132 for just the last hour of TBZ treatment (1 L time point) did not affect c-IAP1 degradation or RIP1 ubiquitination (Figure 3d). Looking specifically at RIP1 in complex-I and the necrosome/complex-II, MG132 pretreatment appeared to stabilize modified RIP1 at complex I and prevent assembly of the necrosome/complex-II (Figure 3e). Therefore, MG132 pretreatment and c-IAP1 stabilization inhibit RIP1 translocation to caspase-8-associated necrosome complex, and consequently necrosome-associated RIP1 ubiquitination.

BV6 treatment caused transient elimination of c-IAP2 in HT29 cells (Figure 3f). Interestingly, the return of TBZ-induced c-IAP2 coincided with the appearance of RIP1 ubiquitination (Figure 3f, compare lanes 3 and 4 with 8 and 9). This increase in c-IAP2 protein abundance at 4 h after TBZ followed increased expression of c-IAP2 messenger RNA (mRNA), raising the possibility that c-IAP2 could promote RIP1 ubiquitination in necroptotic signaling (Supplementary Figure S4C). To determine definitively if c-IAP2 and/or c-IAP1 were responsible for RIP1 ubiquitination in necroptosis, we compared RIP1 modifications in a WT, c-IAP1^−/−^ and c-IAP1^−/−^c-IAP2^−/−^ MEFs. TBZ-induced ubiquitination of RIP1 was comparable in the different MEF lines (Figure 3g), indicating that c-IAP1 and c-IAP2 are dispensable for ubiquitination of RIP1 induced by TBZ. In addition, in the absence of c-IAP1 and c-IAP2, TNF*α* plus zVAD (TZ) treatment was sufficient to trigger RIP1 ubiquitination (Figure 3g). siRNA knockdown of TRAF2, which is the adaptor protein that bridges c-IAPs and RIP1 within complex-I, did not affect TBZ-induced ubiquitination of RIP1 or necroptosis in HT29 cells either (Supplementary Figures S4D and E). Collectively, these data indicate that ubiquitination of RIP1 during necroptosis can occur independently of c-IAPs.

### Upregulation of c-IAP2 in the absence of c-IAP1 decreases necroptosis

**Figure 4:**
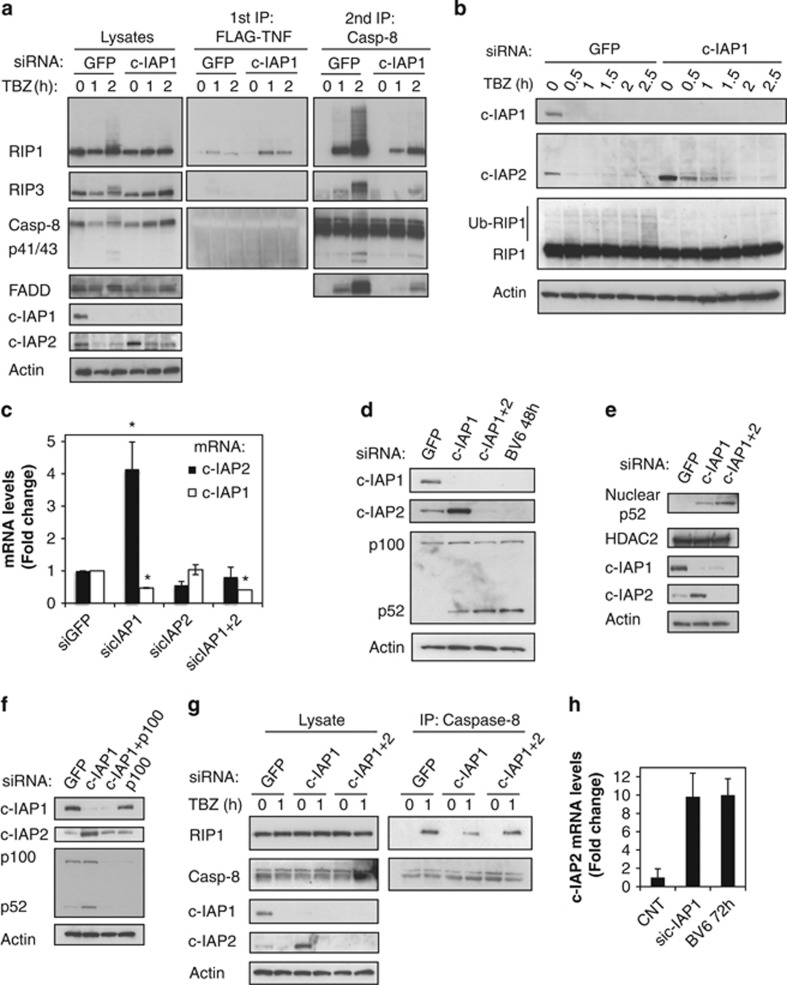
c-IAP1 knockdown inhibits necrosome formation and cell death due to c-IAP2 upregulation. (**a**–**h**) HT29 cells were transfected with the indicated siRNAs or treated with BV6 for 72 or 48 h. (**a**) Cells were treated with Flag-TNF*α* 1 *μ*g/ml (T), BV6 2 *μ*M (B) and zVAD 20 *μ*M (Z) for the indicated periods of time. Cell lysates were first immunoprecipitated with Flag-beads, and the supernatants underwent a second immunoprecipitation with caspase-8 antibody. The pull-downs and lysates were analyzed by western blotting with the indicated antibodies. (**b**) Cells were treated with TNF*α* 20 ng/ml (T), BV6 2 *μ*M (B) and zVAD 20 *μ*M (Z) for the indicated periods of time and cellular lysates were analyzed by western blotting. (**c**) Total mRNA was extracted and c-IAP2 and c-IAP1 mRNA levels were analyzed by quantitative RT-PCR real time. (**d**) Lysates of siRNA-transfected and BV6-treated cells were analyzed by western blotting with the indicated antibodies. (**e**) Nuclear and cytoplasmic extracts of siRNA-transfected cells were analyzed by western blotting with the indicated antibodies. (**f**) Lysates of siRNA-transfected cells were analyzed by western blotting with the indicated antibodies. (**g**) Cells were treated with TNF*α* 20 ng/ml (T), BV6 2 *μ*M (B) and zVAD 20 *μ*M (Z) for the indicated periods of time. Cell lysates were immunoprecipitated with caspase-8 antibody. The pull-downs and lysates were analyzed by western blotting with the indicated antibodies. (**h**) Total mRNA was extracted and c-IAP2 mRNA levels were analyzed as in (**c**)

We were intrigued that knockdown of c-IAP1 in HT29 cells decreased TBZ-induced ubiquitination of RIP1 and necroptotic cell death (Figures 3a and c and Supplementary Figures S4A and B). Analysis of complex-I and the necrosome/complex-II revealed that c-IAP1 knockdown in HT29 cells caused a slight increase in the amount of RIP1 in TBZ-induced complex-I, whereas less RIP1 was incorporated into the caspase-8-containing necrosome/complex-II (Figure 4a). The association of caspase-8 with RIP3 and FADD was also reduced (Figure 4a).

Interestingly, knockdown of c-IAP1 increased the expression of c-IAP2 protein (Figure 4b) and mRNA (Figure 4c), and the increased c-IAP2 protein could not be quickly depleted by BV6 in the absence of c-IAP1 (Figure 4b). Upregulation of c-IAP2 was most likely the result of noncanonical NF-*κ*B activation, as demonstrated by processing of the NF-*κ*B2/p100 transcription factor to p52 (Figure 4d) and accumulation of the p52 subunit in the nucleus (Figure 4e). Indeed, combined knockdown of c-IAP1 and p100 abrogated c-IAP2 upregulation (Figure 4f). Similarly, in KatoIII and Colo201 cells, c-IAP1 knockdown also upregulated c-IAP2 levels and reduced TBZ-induced necroptosis and RIP1 ubiquitination (Supplementary Figures S5A and E). To assess more precisely differences in necrosome formation, HT29 cells were treated with TBZ for 1 h (Figure 4g and Supplementary Figures S6A and B). Although downregulation of c-IAP1 decreased the association of RIP1 with caspase-8, c-IAP2 knockdown had no effect (Supplementary Figure S6A). Elevated c-IAP2 appeared to contribute to the effects of c-IAP1 knockdown on TBZ-induced assembly of the necrosome/complex-II because combined knockdown of c-IAP1 and c-IAP2 resulted in stronger RIP1 necrosome association in comparison with c-IAP1 knockdown (Figure 4g).

Triple knockdown of c-IAP1, c-IAP2 and XIAP triggered a noticeable increase in necrosome formation in agreement with reports that XIAP is a negative regulator of necroptosis (Supplementary Figure S6B).^35^ To assess which combination of IAP knockdowns would mimic BV6 activity in necroptosis, we treated cells with TNF*α* and zVAD, and interestingly, we found that triple knockdown of c-IAP1, c-IAP2 and XIAP had the same effect as BV6 (Supplementary Figure S6C). However, individual downregulation of XIAP did not alter RIP1 necrosome recruitment or TBZ-induced cell death, suggesting that the absence of XIAP exerts strong effects on TBZ-stimulated necrosome formation mostly in the context of c-IAP1/2 loss (Supplementary Figures S6D and E). These results point to an interesting interplay of c-IAPs and IAP antagonists in necroptosis. IAP antagonists eliminate c-IAP proteins to induce necroptosis in HT29 cells, but at the same time, IAP antagonists or c-IAP1 knockdown induce c-IAP2 upregulation owing to noncanonical NF-*κ*B activation. However, the loss of c-IAP1 by knockdown allows the accumulation of c-IAP2, whereas BV6 treatment causes degradation of newly synthetized c-IAP2 (Figures 4d, e and h).

**Figure 5:**
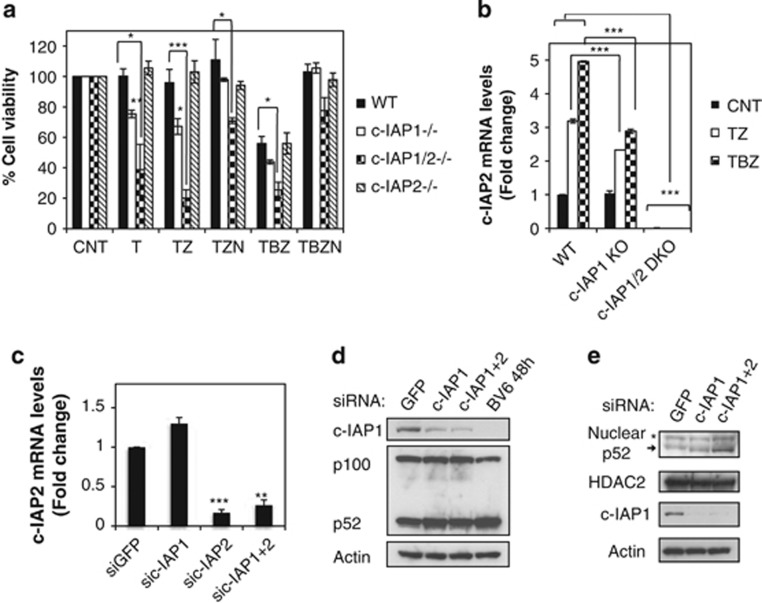
Inability to upregulate c-IAP2 enhances necroptotic cell death. (**a** and **b**) Indicated MEF lines were treated with mTNF*α* 100 ng/ml (T), BV6 2 *μ*M (B), zVAD 20 *μ*M (Z) and Nec-1 30 *μ*M (N). (**a**) Cells were treated 20 h and viability was assessed by CellTiter-Glo. Data are mean±S.E.M. values of at least three experiments. (**b** and **c**). Total mRNA was extracted following 2 h treatment (**b**) or no treatment (**c**) and c-IAP2 mRNA levels were analyzed by quantitative RT-PCR real time. *P<0.05, **P<0.01 and ***P<0.005. (**d**) MEFs were transfected with the indicated siRNAs or treated with BV6 (2*μ*M) for 48 h. Cell lysates were analyzed by western blotting with the indicated antibodies. (**e**) MEFs were transfected with the indicated siRNAs for 48 h. Nuclear and cytoplasmic cellular extracts were analyzed by western blotting with the indicated antibodies. The asterisk denotes a nonspecific band

Next, we investigated the regulation of c-IAP2 levels in MEFs during necroptotic signaling. Both c-IAP1^−/−^ knockout (KO) and c-IAP1/2^−/−^ DKO MEFs were more sensitive to necroptotic cell death compared with wild-type (WT) MEFs (Figure 5a and 3g). Interestingly, c-IAP1^−/−^ KO MEFs did not have elevated levels of c-IAP2 in basal conditions, and c-IAP2 upregulation after necroptotic stimulation was lower compared with that in WT MEFs (Figure 5b). Additionally, transient knockdown of c-IAP1 in MEFs did not significantly affect c-IAP2 mRNA levels (Figure 5c). Contrary to HT29 cells, p100 is constitutively processed to p52 in MEFs and c-IAP1 downregulation caused no significant differences in p100 processing and p52 nuclear translocation (Figures 5d and e). Collectively, these data suggest that the ability to upregulate c-IAP2 determines whether c-IAP1 absence will have positive or negative impact on TNF*α*-induced necroptotic cell death.

### RIP1 undergoes linear ubiquitination in necroptosis

**Figure 6:**
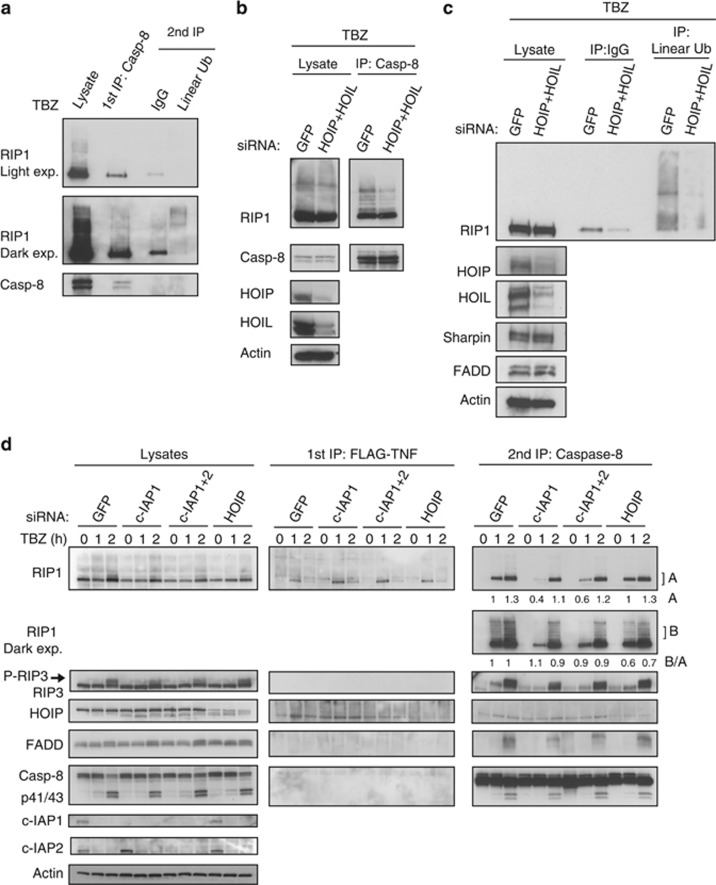
RIP1 undergoes linear ubiquitination upon induction of necroptosis. (**a**–**c**) HT29 cells were pretreated with BV6 2 *μ*M (B) and zVAD 20 *μ*M (Z), and 20 min latter TNF*α* 20 ng/ml (T) was added for another 2 h. (**a**) Cell lysates were first immunoprecipitated with caspase-8 antibody, and the pull-downs were disrupted in 6 M urea and underwent a second immunoprecipitation with linear ubiquitin or control antibody. (**b–d**) HT29 cells were transfected with the indicated siRNAs for 72 h. (**b**) Cell lysates were immunoprecipitated with caspase-8 antibody. (**c**) Cell lysates were immunoprecipitated in 6 M urea with linear ubiquitin or control antibody. (**d**) HT29 cells were pretreated with BV6 2 *μ*M (B) and zVAD 20 *μ*M (Z), and 20 min latter with Flag-TNF*α* 1*μ*g/ml (T) for the indicated periods of time. Cell lysates were first immunoprecipitated with Flag beads, and the supernatants underwent a second immunoprecipitation with caspase-8 antibody. The pull-downs and lysates were analyzed by western blotting with the indicated antibodies. The amount of RIP1 immunoprecipitated by caspase-8 was quantified and indicated below each lane. RIP1 quantification in the upper panel (*A* values) corresponds to the levels of unmodified RIP1 in each lane in comparison with RIP1 levels in GFP 1 h. RIP1 quantification in the lower panel (*B*/*A* values) corresponds to the ratio between the intensity of RIP1 ubiquitination (*B* values) and the levels of co-immunoprecipitated unmodified RIP1 in the upper panel (*A* values)

LUBAC (consisting of HOIP, HOIL1 and sharpin) has been shown to promote linear ubiquitination of RIP1 within the TNFR1 signaling complex.^13, 16^ We examined whether LUBAC mediates RIP1 linear ubiquitination in the necrosome by pretreating cells with BV6 to eliminate c-IAP proteins and thus prevent TNF-mediated recruitment of LUBAC to complex-I.^12, 13^ HT29 cells were treated with TBZ and the necrosome was immunoprecipitated using anticaspase-8 antibody. Necrosome was then disrupted in urea buffer and reimmunoprecipitated with the linear polyubiquitin chain-specific antibody. This demonstrated that necrosome-associated RIP1 is modified with linear polyubiquitin linkages (Figure 6a). To examine the role of LUBAC in necrosome-associated linear polyubiquitination of RIP1, we knocked down HOIP and HOIL1. Downregulation of HOIP and HOIL1 reduced RIP1 ubiquitination in the necrosome (Figure 6b) and necroptosis-specific linear ubiquitination (Figure 6c). HOIP knockdown reduced the levels of caspases-8-bound ubiquitinated RIP1, suggesting that HOIP absence affected RIP1 ubiquitination similarly to c-IAP1 knockdown (RIP1 (dark exposure); Figure 6d). However, quantification of RIP1 levels revealed that c-IAP1 downregulation decreased RIP1 ubiquitination because of the inhibition of necrosome formation (changes in A numbers–unmodified RIP1–but no significant changes in B/A ratio – ubiquitinated over unmodified RIP1) (Figure 6d and Supplementary Figure S7A). On the other hand, HOIP knockdown did not affect necrosome formation (no changes in A numbers) but reduced ubiquitination of RIP1 (lower B/A ratio) (Figure 6d and Supplementary Figure S7B). Knockdown of HOIP did not affect TBZ-stimulated necroptotic cell death either (Supplementary Figures S7C and D). Therefore, c-IAPs and LUBAC regulate RIP1 ubiquitination and necrosome formation through different mechanisms.

We also explored necroptotic signaling in *cpdm* MEFs, which have deficient sharpin expression.^36^ Investigation of linear polyubiquitination of RIP1 during necroptosis revealed no differences between *cpdm* and WT MEFs (Supplementary Figure S8A). As expected, K63-linked RIP1 polyubiquitination was not affected. We verified this result with sharpin knockdown in HT29 cells, and again observed that RIP1 can undergo linear ubiquitination in necroptosis without sharpin (Supplementary Figure S8B). Sharpin knockdown did not affect necrosome formation, even though RIP1 ubiquitination in the TNFR1-associated signaling complex was reduced in the absence of sharpin (Supplementary Figure S8C). In addition, sharpin or HOIP downregulation did not influence each other's protein levels in any of the examined cell lines (Supplementary Figures S8D and F). Therefore, unlike HOIP and HOIL1, sharpin does not seem to be crucial for necrosome-associated RIP1 linear ubiquitination during IAP antagonist-stimulated necroptotic signaling.

## Discussion

Ubiquitination of RIP1 by c-IAP proteins within TNFR1-associated complex-I inhibits RIP1 dissociation from complex-I.^10^ Deubiquitination allows RIP1 to move from the TNFR1-associated complex to the cell death-promoting complex-II or necrosome.^10, 34, 37^ Thus, it is believed that the necrosome contains non-ubiquitinated RIP1. However, contrary to previous reports,^33, 37^ our study shows that RIP1 is ubiquitinated during IAP antagonist and TNF*α*, as well as IAP antagonist and LPS, stimulated necroptosis predominantly with K63 and linear polyubiquitin chains, and that ubiquitinated RIP1 is part of the necrosome complex where it associates with phosphorylated RIP3. Our data show that following deubiquitination, RIP1 leaves the TNFR1-bound complex, only to be ubiquitinated later in the necrosome. Although we do not find conclusive evidence that RIP3 undergoes ubiquitination during necroptosis, it is possible that RIP3 ubiquitination has a role in inflammatory cell death pathways. While our manuscript was in review, another study showed that LPS-driven necroptosis stimulates RIP3 and MLKL ubiquitination.^38^ Given that we used different experimental approaches, it is conceivable that the results from two studies might not completely overlap. Nevertheless, the data from our study and Lawlor *et al.*^38^ shed light on the importance of ubiquitination in necroptotic signaling, which should trigger more investigations of the role of this posttranslational modification in inflammatory cell death pathways.

Given that c-IAPs promote RIP1 ubiquitination within the TNFR1 signaling complex,^8, 11^ we postulated that they regulate necroptosis-associated RIP1 ubiquitination. Further supporting that hypothesis, we observed a decrease in RIP1 ubiquitination after c-IAP1 knockdown and a correlation between c-IAP2 reappearance and RIP1 ubiquitination. However, given that proteasome inhibitor treatment to stabilize c-IAP1 eliminated RIP1 ubiquitination and complete absence of c-IAPs in double KO MEFs did not affect RIP1 ubiquitination, we are fairly certain that c-IAPs are not RIP1 E3 ligases in the necrosome.

Nevertheless, c-IAP1 downregulation inhibits necroptotic cell death, whereas IAP antagonists promote necroptosis. The possible key for understanding this conundrum is c-IAP2, whose levels increase upon c-IAP1 loss because of the activation of noncanonical NF-*κ*B signaling.^18, 39^ Thus, c-IAP2 protein levels may explain the interesting differences between IAP antagonist treatment and c-IAP1 downregulation: specific c-IAP1 loss boosts c-IAP2 levels, whereas BV6 also promotes c-IAP2 upregulation, but ultimately causes its degradation. However, c-IAP2 upregulation is not a global event as in some cell types, such as MEFs, c-IAP1 loss does not activate noncanonical NF-*κ*B signaling nor upregulates c-IAP2 levels. As a consequence, the absence of c-IAP1 does not provide any survival benefit.^40^ Therefore, the ability to activate noncanonical NF-*κ*B signaling and induce c-IAP2 upregulation can determine responsiveness to IAP antagonist-mediated necroptotic cell death. Interestingly, only the triple knockdown of c-IAP1, c-IAP2 and XIAP reproduces BV6-like effects on necrosome formation and necroptotic cell death. Recent studies suggest that XIAP could potentially ubiquitinate necrosome components; however, more studies are needed to decipher the exact role of XIAP in necroptosis.^35, 38, 41^

LUBAC also promotes RIP1 ubiquitination within the TNFR1 signaling complex, but through linear ubiquitin linkages.^13, 16^ We found that necrosome-bound RIP1 is modified with linear ubiquitin chains in an HOIP/HOIL1-dependent manner. Earlier reports suggested that HOIP or HOIL1 knockdown or KO augment TNF*α-*stimulated cell death.^42, 43^ However, we found that in IAP antagonist-pretreated cells linear ubiquitination does not significantly affect necrosome formation or necrotic cell death. IAP antagonist pretreatment inhibits LUBAC recruitment and RIP1 ubiquitination within the TNFR1-associated complex.^13^ Therefore, pretreatment with IAP antagonists dissociates LUBAC function within the necrosome from its role in the TNFR1 signaling complex and explains the differences between the important role HOIP deficiency exerts in TNF*α*-mediated cell death and IAP antagonist-stimulated necroptosis.^15, 16, 17, 44^ Given that downregulation of HOIP and HOIL1 decreases linear ubiquitination of necrosome-bound RIP1 without affecting necrosome formation, linear ubiquitination of RIP1 in necroptotic cells might not be crucial for IAP antagonist-mediated necroptotic cell death.

Sharpin is important for LUBAC activity and the absence of sharpin limits linear ubiquitination in TNFR1 signaling complex and decreases NF-*κ*B signaling.^15, 16, 17^ To our surprise, *cpdm* MEFs or cells with sharpin knockdown showed no decrease in RIP1 linear ubiquitination in necroptosis. In agreement with the reported role of sharpin in TNFR1-mediated signaling,^15, 16, 17^ we observed a decrease in RIP1 ubiquitination in the TNFR1 signaling complex upon sharpin knockdown. Nevertheless, it seems that in the IAP antagonist-stimulated necrosome sharpin is not needed for LUBAC activity. Given that *cpdm* MEFs can form HOIP-HOIL1 complexes with E3 ligase activity, sharpin may be dispensable for LUBAC activity in certain conditions.^16, 17^ In prior studies, *cpdm* mice and cells were found to be sensitive to TNF-induced cell death to various degrees,^15, 16, 44, 45^ which could be explained by the destabilization of HOIP and HOIL1 protein levels in the absence of sharpin.^16, 17, 46^ However, in our study, sharpin absence did not negatively affect the levels of HOIP, and accordingly, IAP antagonist and TNF*α-*driven necrosome-associated RIP1 ubiquitination was also unchanged.

In summary, this study reveals the presence of a second wave of RIP1 ubiquitination during necroptosis and the complex role for c-IAP proteins in the regulation of IAP antagonist-mediated necroptotic cell death. c-IAP1 controls TNF*α*-stimulated necrosome formation without affecting RIP1 ubiquitination. On the other hand, linear RIP1 ubiquitination in the necrosome is mediated by HOIP, although its functional relevance remains elusive. Further studies are needed to fully elucidate the importance of RIP1 ubiquitination in necroptosis.

## Materials and Methods

### Reagents and transfections

The following materials have been used: human recombinant soluble TNF*α* (Genentech, South San Francisco, CA, USA), mouse recombinant soluble TNF*α* (R&D, Minneapolis, MN, USA), Flag-TNF*α* (Enzo, Farmingdale, NY, USA), BV6 (Genentech), z-VAD-Fmk (MBL, Woburn, MA, USA), Nec-1 (Sigma, Atlanta, GA, USA), MG132 (UBPBio, Aurora, CO, USA) and LPS (Invivogen, San Diego, CA, USA). The primary antibodies used were directed against: RIP1, XIAP, TRAF2, FADD (BD Biosciences, San Jose, CA, USA), human and mouse RIP3 (Imgenex, San Diego, CA, USA), human c-IAP1 and mouse pan-c-IAP (R&D), human c-IAP2 (Novus, Littleton, CO, USA), actin, IgG (Santa Cruz, Santa Cruz, CA, USA), ubiquitin (P4D1; Cell Signaling, Danvers, MA, USA), human caspase-8 (Cell signaling and Enzo), mouse caspase-8, mouse c-IAP1 and FLIP (Enzo), human sharpin, p100/p52, HDAC2, IRAK1 (Cell Signaling), K11, K48, K63 and linear ubiquitin chains, and mouse RIP3 (Genentech), HOIP (Aviva Systems, San Diego, CA, USA), HOIL1 (Sigma), mouse sharpin (Proteintech, Chicago, IL, USA), MLKL (Millipore, Billerica, MA, USA) and P-MLKL (Abcam, Cambridge, MA, USA). All siRNA transfections were carried out using Lipofectamine RNAiMax (Invitrogen) for 48 or 72 h; siRNA sequences are included in Supplementary Data.

### Cell lines

Human colon carcinoma HT29, LS411N, Colo201, Colo205, stomach Kato III, Im95m, 23132/87 and mouse L929 cell lines were obtained from the American Type Culture Collection (Manassas, VA, USA). Cells were grown and maintained in 50 : 50 F12 : DMEM medium supplemented with 10% fetal bovine serum, penicillin, streptomycin and 2 mM glutamine at 5% CO_2_. WT and c-IAP1^−/−^, c-IAP1/2^−/−^, c-IAP2^−/−^ and *cpdm* MEFs were cultured in DMEM medium supplemented with 10% heat-inactivated serum, penicillin, streptomycin, 2 mM glutamine and non-essential amino acids at 5%CO_2_. *cpdm* MEFs were kindly provided by John Silke (Walter and Eliza Hall Institute (WEHI), Parkville, VIC, Australia).

### Viability assays

Cell viability was assessed using CellTiter-Glo (Promega) following the manufacturer's specifications. Cell death was monitored by Incucyte Zoom (Essen BioSciences, Ann Harbor, MI, USA) using the cell death dye Sytox Green nucleic acid stain (Life Technologies, Carlsbad, CA, USA) and the dye Nuclear-ID red DNA stain (Enzo) to count the total number of cells.

### Western blot analysis, immunoprecipitation and nuclear extracts

Western blot analyses and immunoprecipitations were performed with the following lysis buffer: 20 mM Tris-HCl (pH 7.5), 150 mM NaCl, 1 mM EDTA, 1% Triton X-100, protease inhibitor cocktail (Roche, Indianapolis, IN, USA), phosStop phosphatase Inhibitor (Roche) and Phosphatase inhibitor cocktail 2 and 3 (Sigma). Cells were lysed on ice for 30 min and centrifuged at 14 000r.p.m. for 10 min at 4 °C.

Immunoprecipitations of RIP1 associated with the TNFR1 complex or the necrosome, 1 × 10^6^ cells were left untreated or treated with FLAG-TNF*α*, BV6 (2 *μ*M) and zVAD for the indicated periods of time. Following PBS washes, cells were lysed in lysis buffer. Cellular lysates were precleared with agarose beads (30 min at 4 °C), and immunoprecipitated with FLAG beads (Sigma) 2 h at 4 °C. The samples were centrifuged 1 min at 1500 r.p.m. to recover the FLAG beads. The supernatants of FLAG immunoprecipitations were reimmunoprecipitated with caspase-8 antibody (Enzo) bound to protein-A/G beads (overnight at 4 °C). Immunoprecipitated protein complexes were washed four times in lysis buffer, eluted by heating in LB sample buffer, resolved on SDS-PAGE and immunoblotted with the indicated antibodies.

Quantification of the intensity of western blot bands was carried out using the Carestream software (New Haven, CT, USA). The amount of RIP1 protein pulled down by caspase-8 immunoprecipitation was quantified and indicated below each lane. RIP1 quantification was expressed as the levels of RIP1 in each sample compared with RIP1 in GFP 1 h. Quantification of RIP1 ubiquitination was measured as the ratio between the intensity of ubiquitinated RIP1 (*B* values) and the levels of co-immunoprecipitated unmodified RIP1 (*A* values).

To assess RIP1 ubiquitination, cell lysates were denatured by boiling in 1% SDS and then diluted 20 times in lysis buffer. Denatured lysates were immunoprecipitated with ubiquitin antibody and protein-A/G beads overnight at 4 °C.

Ubiquitin chain-specific immunoprecipitations were performed by lysing the cells in 6 M urea containing buffer (20 mM Tris-HCl, pH 7.5, 135 mM NaCl, 1.5 mM MgCl_2_, 1 mM EGTA, 1% Triton X-100 and protease inhibitor cocktail; Roche). Linear-specific anti-ubiquitin immunoprecipitations were performed in 6 M urea at room temperature. K11, K48, K63 and IgG immunoprecipitations were performed in 3 M urea at 4 °C as described previously.^47^

For detection of linear-specific ubiquitination of RIP1 within the necrosome, the immunoprecipitated endogenous caspase-8 complexes were washed in lysis buffer and then disrupted in 6 M urea containing buffer and reimmunoprecipitated with linear linkage-specific anti-ubiquitin antibody as described previously.^47^ Immunoprecipitated proteins and lysates were resolved on SDS-PAGE and immunoblotted with the indicated antibodies. For total RIP1 linear ubiquitination, the cells were lysed directly in 6 M urea buffer.

Nuclear extracts were obtained with the NE-PER Nuclear and Cytoplasmic Extraction Reagent Kit Pierce (Chicago, IL, USA) following the manufacturer's recommendations.

Dephosphorylation of RIP3 was performed by treating cellular lysates with λ-phosphatase at 30 °C for 2 h.

### Real-time quantitative PCR

RNA was isolated from cells with the RNeasy Mini Kit (Qiagen, Valencia, CA, USA) following standard protocols. An on-column DNase treatment was included. cDNA was generated from each RNA sample using a Taqman Gene Expression Cells to Ct Kit (Life Technologies). Gene expression assays were synthesized in-house: RPL19-FW, 5′-AGCGGATTCTCATGGAACA-3′ and RPL19-Rv, 5′-CTGGTCAGCCAGGAGCTT-3′ RPL19-Probe, 5′-FAM-TCCACAAGCTGAAGGCAGACAAGG-TAMRA-3′ hcIAP1-FW, 5′-TGTTTGGTGAACTATATTAGTATGTATGTGTACC-3′ and hcIAP1-Rv, 5′-GAAAGAACAACAAATCCAGTAACTCCT-3′ hcIAP1-Probe,5′-FAM-AAGGGAGTAGTGTCACTGCTTGTTATGCATCATTT-TAMRA-3′ or from Life Technologies: hcIAP2-Hs00985031_g1, mcIAP2-Mm00431800_m1 and mGAPDH-Mm99999915_m1. Target gene levels were normalized against RPL19 or GAPDH gene expression.

### Statistic analysis

Data are presented as the mean±S.E.M. Statistical analysis was performed using Student's *t*-test. Results were considered significant if *P<0.05, **P<0.01 or ***P<0.005.

## Abbreviations

RIP1: receptor-interacting protein 1
RIP3: receptor-interacting protein 3
TNF-*α*: Tumor necrosis factor-*α*
TNFR1: TNF receptor 1
IAP: inhibitor of apoptosis
c-IAP: cellular inhibitor of apoptosis
XIAP: X chromosome-linked IAP
LUBAC: linear ubiquitin chain assembly complex
MLKL: mixed lineage kinase domain-like
MEF: mouse embryonic fibroblasts
IP: immunoprecipitation

## References

[bib1] Hanahan D, Weinberg RA. Hallmarks of cancer: the next generation. Cell 2011; 144: 646–674. 21376230 10.1016/j.cell.2011.02.013

[bib2] Linkermann A, Green DR. Necroptosis. N Engl J Med 2014; 370: 455–465. 24476434 10.1056/NEJMra1310050 PMC4035222

[bib3] Steller H. Mechanisms and genes of cellular suicide. Science 1995; 267: 1445–1449. 7878463 10.1126/science.7878463

[bib4] Zong WX, Thompson CB. Necrotic death as a cell fate. Genes Dev 2006; 20: 1–15. 16391229 10.1101/gad.1376506

[bib5] Kaiser WJ, Upton JW, Mocarski ES. Viral modulation of programmed necrosis. Curr Opin Virol 2013; 3: 296–306. 23773332 10.1016/j.coviro.2013.05.019 PMC3821070

[bib6] Vanden Berghe T, Linkermann A, Jouan-Lanhouet S, Walczak H, Vandenabeele P. Regulated necrosis: the expanding network of non-apoptotic cell death pathways. Nat Rev Mol Cell Biol 2014; 15: 135–147. 24452471 10.1038/nrm3737

[bib7] Silke J, Brink R. Regulation of TNFRSF and innate immune signalling complexes by TRAFs and cIAPs. Cell Death Differ 2010; 17: 35–45. 19680262 10.1038/cdd.2009.114

[bib8] Dynek JN, Goncharov T, Dueber EC, Fedorova AV, Izrael-Tomasevic A, Phu L et al. c-IAP1 and UbcH5 promote K11-linked polyubiquitination of RIP1 in TNF signalling. EMBO J 2010; 29: 4198–4209. 21113135 10.1038/emboj.2010.300 PMC3018797

[bib9] Goncharov T, Niessen K, de Almagro MC, Izrael-Tomasevic A, Fedorova AV, Varfolomeev E et al. OTUB1 modulates c-IAP1 stability to regulate signalling pathways. EMBO J 2013; 32: 1103–1114. 23524849 10.1038/emboj.2013.62 PMC3630360

[bib10] Bertrand MJ, Milutinovic S, Dickson KM, Ho WC, Boudreault A, Durkin J et al. cIAP1 and cIAP2 facilitate cancer cell survival by functioning as E3 ligases that promote RIP1 ubiquitination. Mol Cell 2008; 30: 689–700. 18570872 10.1016/j.molcel.2008.05.014

[bib11] Varfolomeev E, Goncharov T, Fedorova AV, Dynek JN, Zobel K, Deshayes K et al. c-IAP1 and c-IAP2 are critical mediators of tumor necrosis factor alpha (TNFalpha)-induced NF-kappaB activation. J Biol Chem 2008; 283: 24295–24299. 18621737 10.1074/jbc.C800128200 PMC3259840

[bib12] Varfolomeev E, Goncharov T, Maecker H, Zobel K, Kömüves LG, Deshayes K et al. Cellular inhibitors of apoptosis are global regulators of NF-kappaB and MAPK activation by members of the TNF family of receptors. Sci Signal 2012; 5: ra22. 22434933 10.1126/scisignal.2001878

[bib13] Haas TL, Emmerich CH, Gerlach B, Schmukle AC, Cordier SM, Rieser E et al. Recruitment of the linear ubiquitin chain assembly complex stabilizes the TNFR1 signaling complex and is required for TNF-mediated gene induction. Mol Cell 2009; 36: 831–844. 20005846 10.1016/j.molcel.2009.10.013

[bib14] Kirisako T, Kamei K, Murata S, Kato M, Fukumoto H, Kanie M et al. A ubiquitin ligase complex assembles linear polyubiquitin chains. EMBO J 2006; 25: 4877–4887. 17006537 10.1038/sj.emboj.7601360 PMC1618115

[bib15] Ikeda F, Deribe YL, Skånland SS, Stieglitz B, Grabbe C, Franz-Wachtel M et al. SHARPIN forms a linear ubiquitin ligase complex regulating NF-kappaB activity and apoptosis. Nature 2011; 471: 637–641. 21455181 10.1038/nature09814 PMC3085511

[bib16] Gerlach B, Cordier SM, Schmukle AC, Emmerich CH, Rieser E, Haas TL et al. Linear ubiquitination prevents inflammation and regulates immune signalling. Nature 2011; 471: 591–596. 21455173 10.1038/nature09816

[bib17] Tokunaga F, Nakagawa T, Nakahara M, Saeki Y, Taniguchi M, Sakata S et al. SHARPIN is a component of the NF-kappaB-activating linear ubiquitin chain assembly complex. Nature 2011; 471: 633–636. 21455180 10.1038/nature09815

[bib18] Varfolomeev E, Blankenship JW, Wayson SM, Fedorova AV, Kayagaki N, Garg P et al. IAP antagonists induce autoubiquitination of c-IAPs, NF-kappaB activation, and TNFalpha-dependent apoptosis. Cell 2007; 131: 669–681. 18022362 10.1016/j.cell.2007.10.030

[bib19] Vince JE, Wong WW, Khan N, Feltham R, Chau D, Ahmed AU et al. IAP antagonists target cIAP1 to induce TNFalpha-dependent apoptosis. Cell 2007; 131: 682–693. 18022363 10.1016/j.cell.2007.10.037

[bib20] Moulin M, Anderton H, Voss AK, Thomas T, Wong WW, Bankovacki A et al. IAPs limit activation of RIP kinases by TNF receptor 1 during development. EMBO J 2012; 31: 1679–1691. 22327219 10.1038/emboj.2012.18 PMC3321198

[bib21] Fulda S, Vucic D. Targeting IAP proteins for therapeutic intervention in cancer. Nat Rev Drug Discov 2012; 11: 109–124. 22293567 10.1038/nrd3627

[bib22] Cho YS, Challa S, Moquin D, Genga R, Ray TD, Guildford M et al. Phosphorylation-driven assembly of the RIP1–RIP3 complex regulates programmed necrosis and virus-induced inflammation. Cell 2009; 137: 1112–1123. 19524513 10.1016/j.cell.2009.05.037 PMC2727676

[bib23] Degterev A, Hitomi J, Germscheid M, Ch'en IL, Korkina O, Teng X et al. Identification of RIP1 kinase as a specific cellular target of necrostatins. Nat Chem Biol 2008; 4: 313–321. 18408713 10.1038/nchembio.83 PMC5434866

[bib24] Newton K, Dugger DL, Wickliffe KE, Kapoor N, de Almagro MC, Vucic D, Komuves L et al. Activity of protein kinase RIPK3 determines whether cells die by necroptosis or apoptosis. Science 2014; 343: 1357–1360. 24557836 10.1126/science.1249361

[bib25] Polykratis A, Hermance N, Zelic M, Roderick J, Kim C, Van TM et al. Cutting edge: RIPK1 Kinase inactive mice are viable and protected from TNF-induced necroptosis *in vivo*. J Immunol 2014; 193: 1539–1543. 25015821 10.4049/jimmunol.1400590 PMC4119562

[bib26] Wang H, Sun L, Su L, Rizo J, Liu L, Wang LF et al. Mixed lineage kinase domain-like protein MLKL causes necrotic membrane disruption upon phosphorylation by RIP3. Mol Cell 2014; 54: 133–146. 24703947 10.1016/j.molcel.2014.03.003

[bib27] Cai Z, Jitkaew S, Zhao J, Chiang HC, Choksi S, Liu J et al. Plasma membrane translocation of trimerized MLKL protein is required for TNF-induced necroptosis. Nat Cell Biol 2014; 16: 55–65. 24316671 10.1038/ncb2883 PMC8369836

[bib28] Zhao J, Jitkaew S, Cai Z, Choksi S, Li Q, Luo J et al. Mixed lineage kinase domain-like is a key receptor interacting protein 3 downstream component of TNF-induced necrosis. Proc Natl Acad Sci USA 2012; 109: 5322–5327. 22421439 10.1073/pnas.1200012109 PMC3325682

[bib29] Sun L, Wang H, Wang Z, He S, Chen S, Liao D et al. Mixed lineage kinase domain-like protein mediates necrosis signaling downstream of RIP3 kinase. Cell 2012; 148: 213–227. 22265413 10.1016/j.cell.2011.11.031

[bib30] Dondelinger Y, Declercq W, Montessuit S, Roelandt R, Goncalves A, Bruggeman I et al. MLKL compromises plasma membrane integrity by binding to phosphatidylinositol phosphates. Cell Rep 2014; 7: 971–981. 24813885 10.1016/j.celrep.2014.04.026

[bib31] Kaczmarek A, Vandenabeele P, Krysko DV. Necroptosis: the release of damage-associated molecular patterns and its physiological relevance. Immunity 2013; 38: 209–223. 23438821 10.1016/j.immuni.2013.02.003

[bib32] Vucic D, Dixit VM, Wertz IE. Ubiquitylation in apoptosis: a post-translational modification at the edge of life and death. Nat Rev Mol Cell Biol 2011; 12: 439–452. 21697901 10.1038/nrm3143

[bib33] Moquin DM, McQuade T, Chan FK. CYLD deubiquitinates RIP1 in the TNFalpha-induced necrosome to facilitate kinase activation and programmed necrosis. PLoS One 2013; 8: e76841. 24098568 10.1371/journal.pone.0076841 PMC3788787

[bib34] Ofengeim D, Yuan J. Regulation of RIP1 kinase signalling at the crossroads of inflammation and cell death. Nat Rev Mol Cell Biol 2013; 14: 727–736. 24129419 10.1038/nrm3683

[bib35] Yabal M, Müller N, Adler H, Knies N, Groß CJ, Damgaard RB et al. XIAP restricts TNF- and RIP3-dependent cell death and inflammasome activation. Cell Rep 2014; 7: 1796–1808. 24882010 10.1016/j.celrep.2014.05.008

[bib36] Seymour RE, Hasham MG, Cox GA, Shultz LD, Hogenesch H, Roopenian DC et al. Spontaneous mutations in the mouse Sharpin gene result in multiorgan inflammation, immune system dysregulation and dermatitis. Genes Immun 2007; 8: 416–421. 17538631 10.1038/sj.gene.6364403

[bib37] O'Donnell MA, Perez-Jimenez E, Oberst A, Ng A, Massoumi R, Xavier R et al. Caspase 8 inhibits programmed necrosis by processing CYLD. Nat Cell Biol 2011; 13: 1437–1442. 22037414 10.1038/ncb2362 PMC3229661

[bib38] Lawlor KE, Khan N, Mildenhall A, Gerlic M, Croker BA, D'Cruz AA et al. RIPK3 promotes cell death and NLRP3 inflammasome activation in the absence of MLKL. Nat Commun 2015; 6: 6282. 25693118 10.1038/ncomms7282 PMC4346630

[bib39] McComb S, Cheung HH, Korneluk RG, Wang S, Krishnan L, Sad S. cIAP1 and cIAP2 limit macrophage necroptosis by inhibiting Rip1 and Rip3 activation. Cell Death Differ 2012; 19: 1791–1801. 22576661 10.1038/cdd.2012.59 PMC3469059

[bib40] Vanlangenakker N, Vanden Berghe T, Bogaert P, Laukens B, Zobel K, Deshayes K et al. cIAP1 and TAK1 protect cells from TNF-induced necrosis by preventing RIP1/RIP3-dependent reactive oxygen species production. Cell Death Differ 2011; 18: 656–665. 21052097 10.1038/cdd.2010.138 PMC3131911

[bib41] Wong WW, Vince JE, Lalaoui N, Lawlor KE, Chau D, Bankovacki A et al. cIAPs and XIAP regulate myelopoiesis through cytokine production in an RIPK1- and RIPK3-dependent manner. Blood 2014; 123: 2562–2572. 24497535 10.1182/blood-2013-06-510743

[bib42] Vanlangenakker N, Bertrand MJ, Bogaert P, Vandenabeele P, Vanden Berghe T. TNF-induced necroptosis in L929 cells is tightly regulated by multiple TNFR1 complex I and II members. Cell Death Dis 2011; 2: e230. 22089168 10.1038/cddis.2011.111 PMC3223695

[bib43] Peltzer N, Rieser E, Taraborrelli L, Draber P, Darding M, Pernaute B et al. HOIP deficiency causes embryonic lethality by aberrant TNFR1. Cell Rep 2014; 9: 153–165. 25284787 10.1016/j.celrep.2014.08.066

[bib44] Kumari S, Lopez-Mosqueda J, Shiraishi R, Romanowska M, Lutzmayer S, Kuiper J et al. *S*harpin prevents skin inflammation by inhibiting TNFR1-induced keratinocyte apoptosis. Elife 2014; 3: e03422. 10.7554/eLife.03422 PMC4225491 25443631

[bib45] Rickard JA, Anderton H, Etemadi N, Nachbur U, Darding M, Peltzer N et al. TNFR1-dependent cell death drives inflammation in Sharpin-deficient mice. Elife 2014; 3: e03464. 10.7554/eLife.03464 PMC4270099 25443632

[bib46] Tamiya H, Terao M, Takiuchi T, Nakahara M, Sasaki Y, Katayama I et al. IFN-gamma or IFN-alpha ameliorates chronic proliferative dermatitis by inducing expression of linear ubiquitin chain assembly complex. J Immunol 2014; 192: 3793–3804. 24634492 10.4049/jimmunol.1302308

[bib47] Newton K, Matsumoto ML, Wertz IE, Kirkpatrick DS, Lill JR, Tan J et al. Ubiquitin chain editing revealed by polyubiquitin linkage-specific antibodies. Cell 2008; 134: 668–678. 18724939 10.1016/j.cell.2008.07.039

