# Supplementary material for: Cellular IAP proteins and LUBAC differentially regulate necrosome-associated RIP1 ubiquitination
Source: Cell Death Dis. 2015 Jun 25;6(6):e1800–. doi: 10.1038/cddis.2015.158 (PMC4669837; doi:10.1038/cddis.2015.158)

Cellular IAP proteins and LUBAC differentially regulate necrosome associated RIP1 ubiquitination

M. Cristina de Almagro[1], Tatiana Goncharov[1], Kim Newton[2], Domagoj Vucic[1*]

Supplemental materials and methods

siRNA used

The siRNAs sense sequences used:

- Luc: GTATCTCTTCATAGCCTTATT
- GFP: GGCACGCCAGGAGCGCACCTT
- hcIAP1-10: AAAGAGAGCCATTCTGTTCTT
- hcIAP1-12: TCGCAATGATGATGTCAAATT
- hcIAP2-25: TCTAACACAAGATCATTGATT
- hcIAP2-29: ATTCGGTACAGTTCACATGTT
- mcIAP1: GCAAGTGCTGGATTCTATTTT
- mcIAP2: GCACAAGTCCCTACCACTTTT
- XIAP-14: GTAGATAGATGGCAATATGTT
- XIAP-15: GAACTGGGCAGGTTGTAGATT
- XIAP-16: GAAAGAGATTAGTACTGAATT
- XIAP-17: GGACTCTACTACACAGGTATT
- TRAF2-3: GTGTCGAGTCCCTTGCAGATT
- TRAF2-4: GGTCTTGGAGATGGAGGCATT
- TRAF2-7: CGACGTGACTTCATCCTCTTT
- HOIP-6: GGC GTG GTG TCA AGT TTA ATT
- HOIP-8: GCA GAA TAC TCA TCC AAG ATT
- HOIL1-3: CGA CAG AGA TGC TGA AGG TTT
- HOIL1-4: GAC CAA GAA AGC AGA GGA ATT
- Sharpin-2: CCACCCAGCACGAGCTACATT
- Sharpin-3: GGTCACACTTGAAGACGCTTT
- Sharpin-9: CCGCAGTGCTCTTGGCTGTTT
- p100-5: CGAACAGCCTTGCATCTAGTT
- p100-7: AGACGAGTGTGGTGAGCTTTT

Supplementary figure legends

Figure S1. ***RIP1 ubiquitination.*** **A.** RIP1 ubiquitination requires complete necroptosis induction. HT29 cells were treated for 3h with BV6 2μM (B), zVAD 20μM (Z) or Nec-1 30μM (N). Cell lysates were analyzed by western blotting with the indicated antibodies. **B.** RIP3 dephosphorylation. Following treatment with TBZ for 3 h, cellular lysates were left untreated or treated with λ-phosphatase. **C.** L929 cells were treated for 3h with TNFα 10ng/ml (T) and zVAD 20μM (Z). Cell lysates were analyzed by western blotting with the indicated antibodies. **D.** The indicated cell lines were treated with TNFα 20ng/ml (T), BV6 2μM (B) and zVAD

20µM (Z) for 2h. Cell lysates were immunoprecipitated with Caspase-8 antibody. The pull-downs and lysates were analyzed by western blotting with the indicated antibodies.

Figure S2. ***RIP1 undergoes K63 and linear chain-linked polyubiquitination during necroptosis but not apoptosis.*** **A.** Colo205 and 23132/87 cells were treated with TNFα 20ng/ml (T), BV6 2µM (B) and zVAD 20µM (Z) for 2h. Cells were lysed in 6M urea buffer and immunoprecipitated using linkage-specific anti-ubiquitin antibodies or control antibody. Immunoprecipitated proteins were detected using indicated antibodies. **B.** HT29 cells were not treated or treated with TNFα 20ng/ml (T) and BV6 2µM (B) for 3.5 hours. Cells were lysed in 6M urea buffer and immunoprecipitated using linkage-specific anti-ubiquitin antibodies or control antibody. Immunoprecipitated proteins were detected using indicated antibodies. **C.** MEF cells were not treated or treated with TNFα (100 ng/ml), BV6 (2 µM) and zVAD (20µM) for 2 hours. Cells were lysed and immunoprecipitated as in (A) and immunoprecipitated proteins were detected using indicated antibodies.

Figure S3. ***RIP1 undergoes K63 and linear chain-linked polyubiquitination during LPS and BV6-induced necroptosis. A*.** L929 cells were treated with TNFα 10ng/ml (T), BV6 2µM (B), LPS (200ng/ml) and zVAD 20µM (Z) for 20h and cell viability was assessed by CellTiter-Glo. Data are mean ± SEM values of two experiments. **B.** L929 cells were not treated or treated with TNFα 10ng/ml (T) and zVAD 20µM (Z), LPS (200 ng/ml) BV6 2µM (B) and zVAD 20µM (Z) for 3 hours, or LPS alone for 1 hour. Cells were lysed in 6M urea buffer and immunoprecipitated using linkage-specific anti-ubiquitin antibodies or control antibody. Immunoprecipitated proteins were detected using indicated antibodies. **C.** L929 cells were not treated or treated with TNFα 10ng/ml (T) and zVAD 20µM (Z), LPS 200 ng/ml (L) and zVAD 20µM (Z), or LPS 200 ng/ml (L), BV6 2µM (B) and zVAD 20µM (Z) for 3 hours. Cells were lysed and immunoprecipitated as in (**B**) and immunoprecipitated proteins were detected using indicated antibodies. Asterix in panels B and C denotes unspecific cross-reacting band, while arrowhead points to the IRAK1 protein.

Figure S4. ***Involvement of c-IAPs and TRAF2 in necroptotic cell death.*** **A-E.** HT29 cells were treated with TNFα 20ng/ml (T), BV6 2µM (B) and zVAD 20µM (Z). **A.** Cell death was assessed by Sytox positive cells. Values were normalized to the total number of cells measured by Nuclear-ID red DNA. Data are mean ± SEM values of three experiments. **B.** c-IAPs regulate necroptotic cell death. HT29 cells were transfected with the indicated siRNAs. After 72h cells were left untreated or treated for 4h. Phase or Sytox images were acquired with an Incucyte Zoom. **C.** c-IAP2 upregulation in necroptotic cells. HT29 cells were treated for the indicated periods of time. Total mRNA was extracted and c-IAP2 mRNA levels were analyzed by quantitative RT-PCR Real time. Data are mean ± SEM values of two experiments. **D-E.** TRAF2 does not regulate RIP1

ubiquitination or cell death during necroptosis. **D.** HT29 cells were transfected with the indicated siRNAs for 72h, and then treated for 3h. Cell lysates were analyzed by western blotting with the indicated antibodies. **E.** HT29 cells were treated for 20h and cell viability was assessed by CellTiter-Glo. Data are mean ± SEM values of seven experiments.

Figure S5. ***Regulation of necroptosis by c-IAP1 and c-IAP2.*** KatoIII **(A, B)** or Colo201 **(C-E)** cells were transfected with the indicated siRNAs for 72h and then treated with TNFα 20ng/ml (T), BV6 2µM (B) and/or zVAD 20µM (Z). **A, C.** Cells were treated for 20h as indicated and cell viability was assessed by CellTiter-Glo. Data are mean ± SEM values of three experiments. **D.** Cell death was measured by Sytox positive cells. Values were normalized to the total number of cells measured by Nuclear-ID red DNA. Data are mean ± SEM values of three experiments. **B, E.** Cells were treated for 3h as indicated and cellular lysates were analyzed by western blotting with the indicated antibodies.

Figure S6. ***The role of IAP proteins in necrosome formation.*** **A-E.** HT29 cells were transfected with the indicated siRNAs. 72h after transfection cells were treated with necroptotic stimulus. **A.** Cells were treated with TNFα 20ng/ml (T), BV6 2µM (B) and zVAD 20µM (Z) for 1 hour. Cell lysates were immunoprecipitated with Caspase-8 antibody. The pull-downs and lysates were analyzed by western blotting with the indicated antibodies. **B.** Cells were treated with Flag-TNFα 1µg/ml (T), BV6 2µM (B) and zVAD 20µM (Z) for 1 hour. Cell lysates were first immunoprecipitated with Flag-beads, and the supernatants underwent a second immunoprecipitation with Caspase-8 antibody. The pull-downs and lysates were analyzed by western blotting with the indicated antibodies. **C.** HT29 were transfected with the indicated siRNAs or treated with BV6 2µM (B). After 72h, cells were treated for 20h with TNFα 20ng/ml (T) and zVAD 20µM (Z). Cell viability was assessed by CellTiter-Glo. Data are mean ± SEM values of three experiments. **D.** Cells were treated with Flag-TNFα 1µg/ml (T), BV6 2µM (B) and zVAD 20µM (Z) for the indicated period of time. Cell lysates were first immunoprecipitated with Flag-beads, and the supernatants underwent a second immunoprecipitation with Caspase-8 antibody. The pull-downs and lysates were analyzed by western blotting with the indicated antibodies. **E.** HT29 were transfected with the indicated siRNAs. After 72h, cells were treated for 20h with TNFα 20ng/ml (T), BV6 2µM (B), zVAD 20µM (Z) and Nec-1 30µM (N). Cell viability was assessed by CellTiter-Glo. Data are mean ± SEM values of five experiments.

Figure S7. ***HOIP downregulation does not affect necroptotic cell death.*** **A and B.** Quantification of RIP1 co-immunoprecipitated with caspase-8. **A.** Unmodified RIP1 levels co-immunoprecipitated caspase-8 at 1h time point (A values from Figure 6D). **B.** Amount of ubiquitinated RIP1 normalized to the levels of unmodified RIP1 co-immunoprecipitated at 1h time point (B/A values from Figure 6D). **C and D.** HT29 (**C**) and Colo201 (**D**) cells were transfected with the indicated siRNAs. After 72 h cells were pre-treated with BV6 2µM (B) and zVAD

20μM (Z) for 20min after which TNFα 20ng/ml (T) was added for 20h. Cell viability was assessed by CellTiter-Glo. Data are mean ± SEM values of three experiments.

Figure S8. ***Sharpin is dispensable for necrosome-associated RIP1 linear ubiquitination.*** **A.** Wild type or *cpdm* MEFs were pre-treated with BV6 2μM (B) and zVAD 20μM (Z) for 20min after which mTNFα 100ng/ml (T) was added. Cells were treated for 2h and cellular lysates were immunoprecipitated in 6M urea with the indicated antibodies. **B-D.** HT29 cells were transfected with the indicated siRNAs. After 72h cells were pre-treated with BV6 2μM (B) and zVAD 20μM (Z) for 20min after which TNFα 20ng/ml (T) was added. **B.** HT29 cells were treated for 2h and cellular lysates were immunoprecipitated in 6M urea with the indicated antibodies. **C.** HT29 cells were treated with Flag-TNFα 1μg/ml (T), B and Z for the indicated periods of time. Cell lysates were first immunoprecipitated with Flag-beads, and the supernatants underwent a second immunoprecipitation with Caspase-8 antibody. **D, E and F.** Sharpin and HOIP knockdowns do not affect each other's protein stability. HT29 (**D**), LS411N (**E**) or Colo201 (**F**) cells were transfected with the indicated siRNAs. After 72 h cells were pre-treated with BV6 2μM (B) and zVAD 20μM (Z) for 20min after which TNFα 20ng/ml (T) was added for 2h. Cell lysates were analyzed by western blotting with the indicated antibodies.

Figure S1, de Almagro et al.

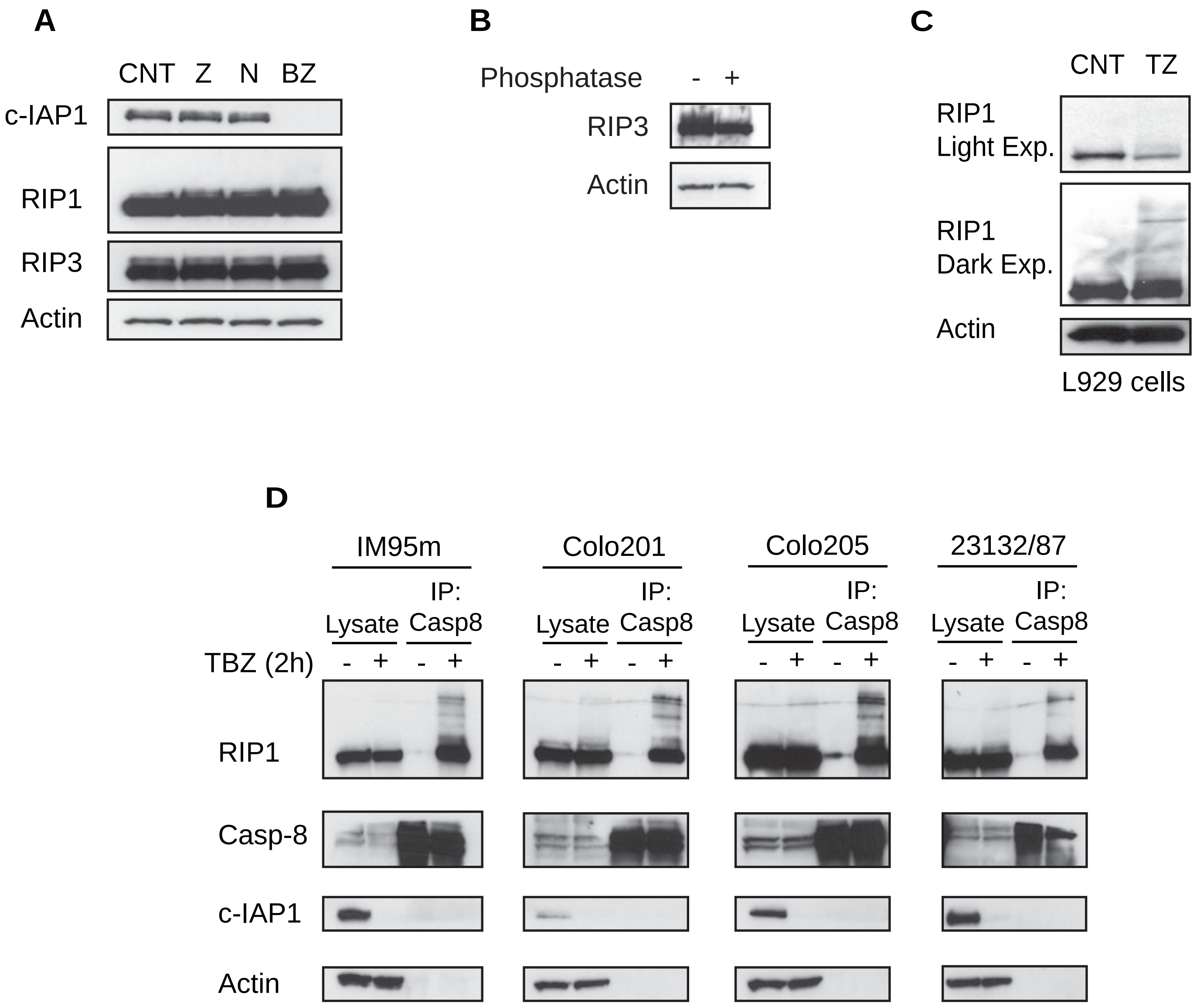

Figure S2, de Almagro et al

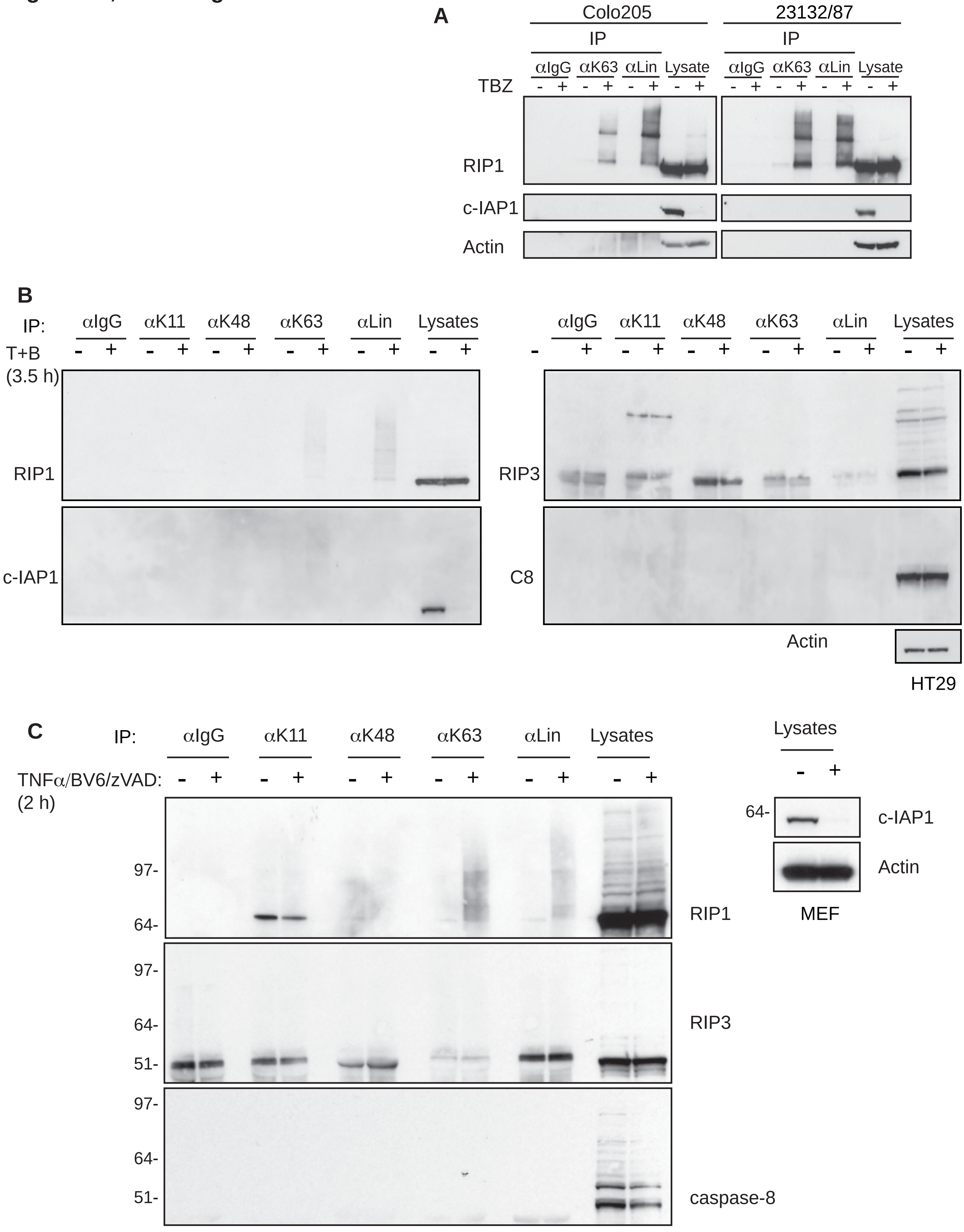

Figure S3, de Almagro et al.

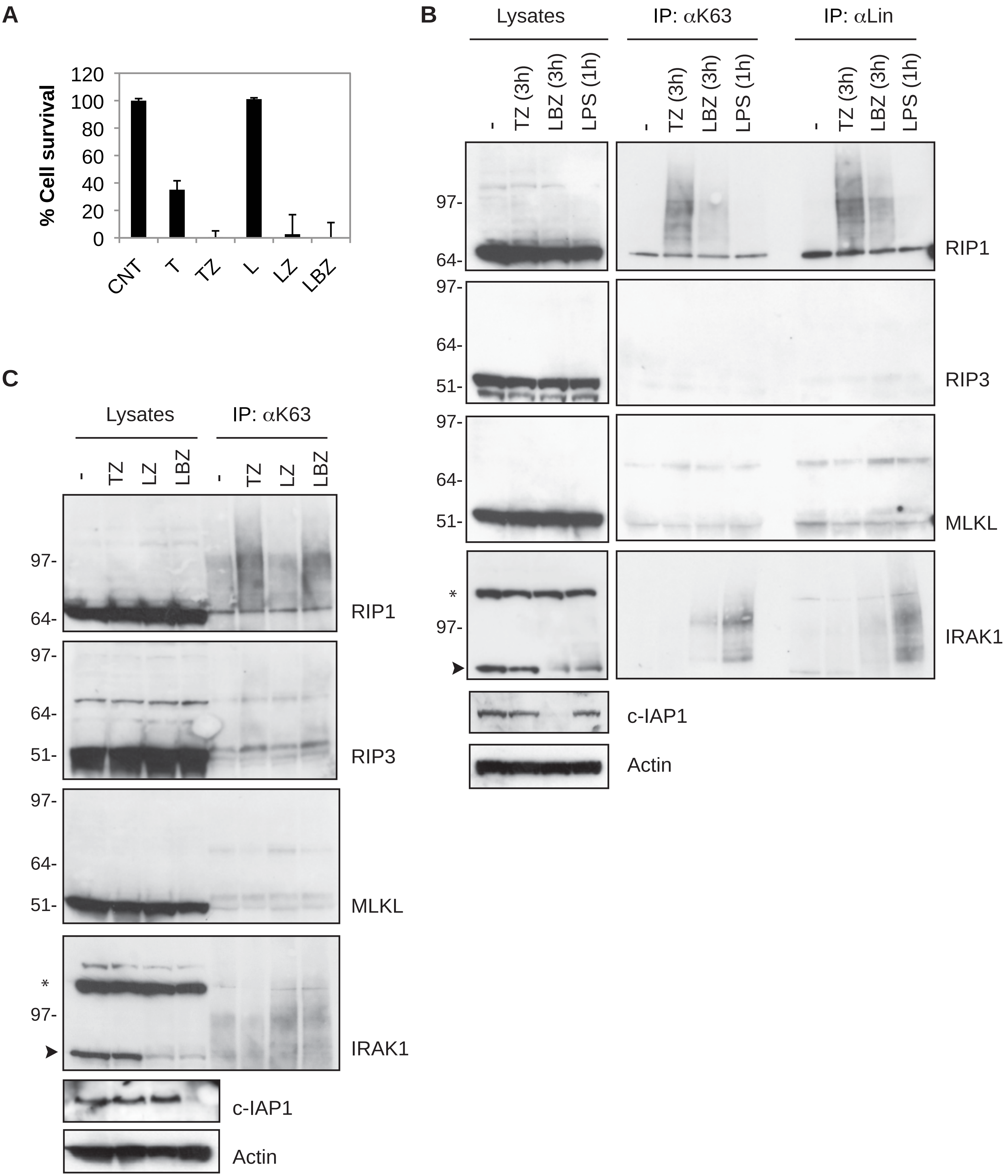

Figure S4, de Almagro et al.

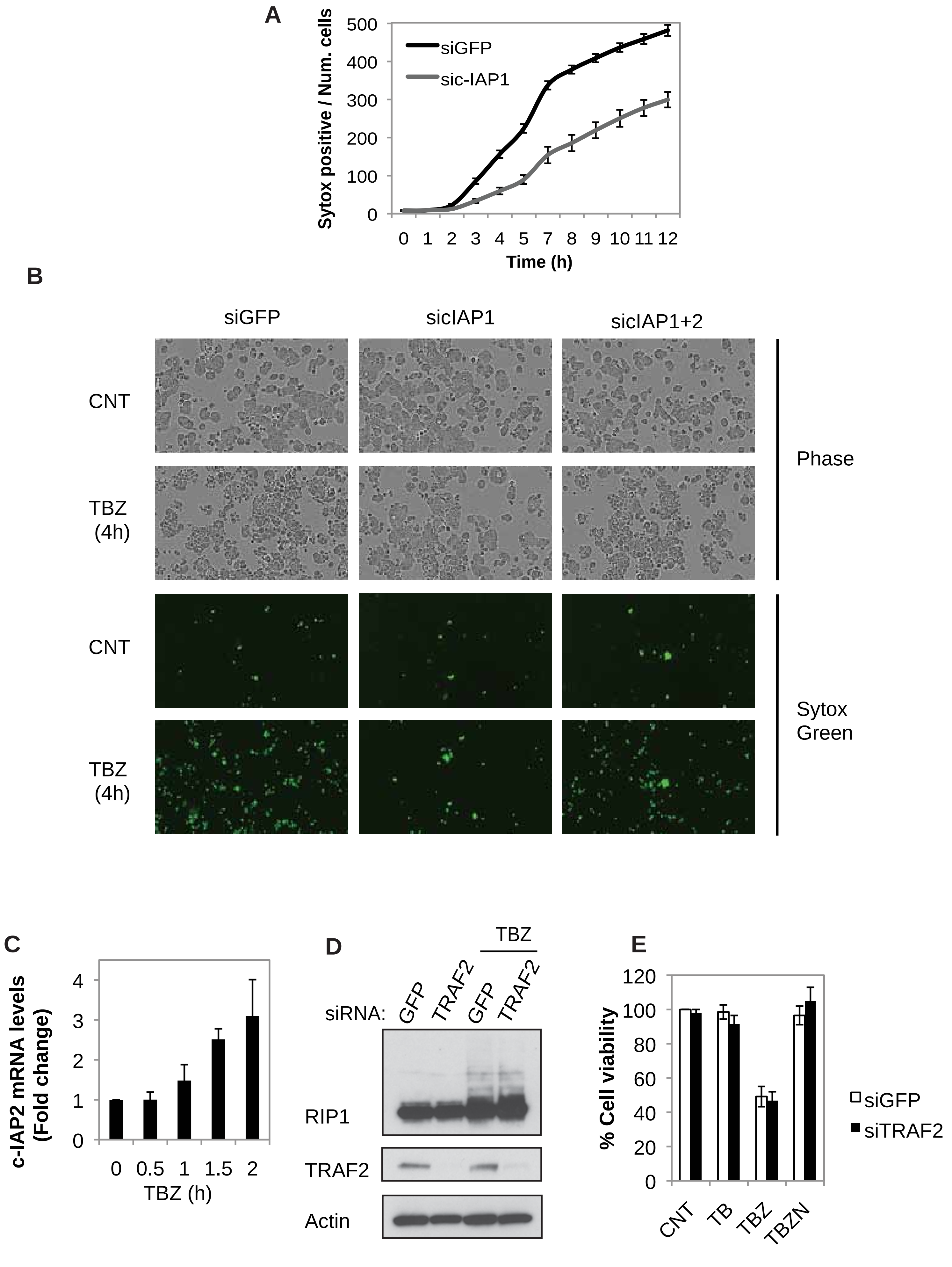

Figure S5, de Almagro et al.

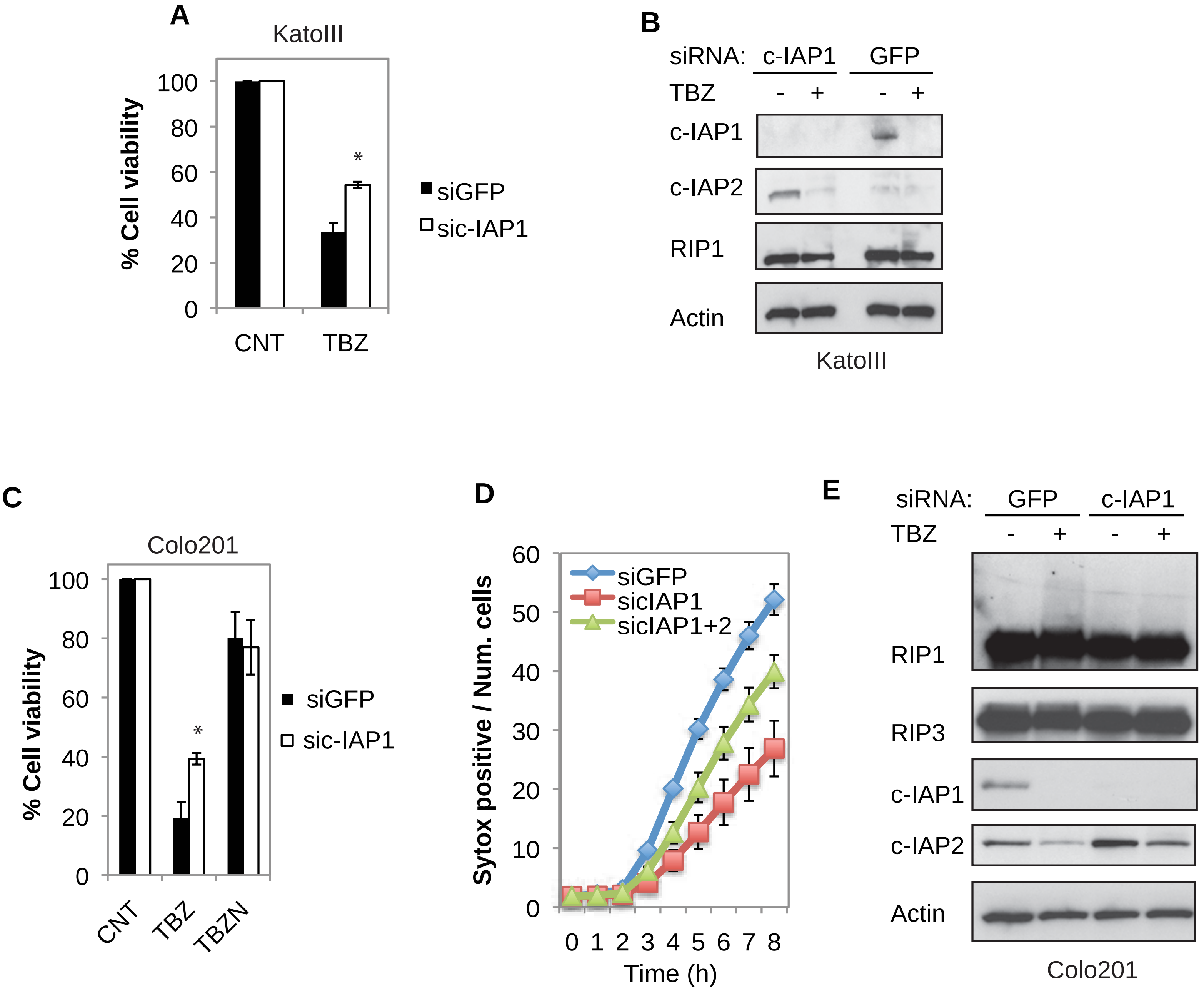

Figure S6, de Almagro et al.

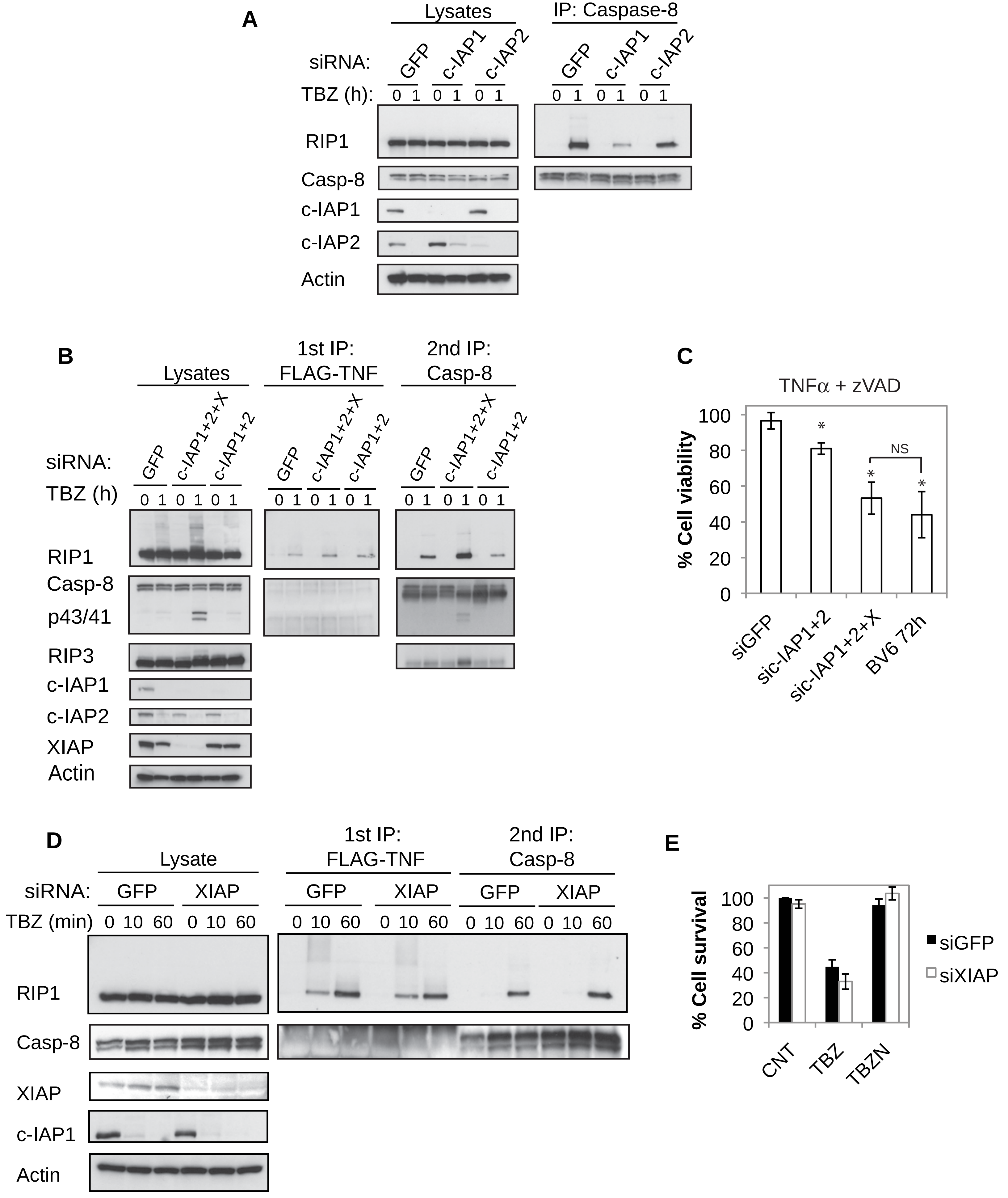

Figure S7, de Almagro et al.

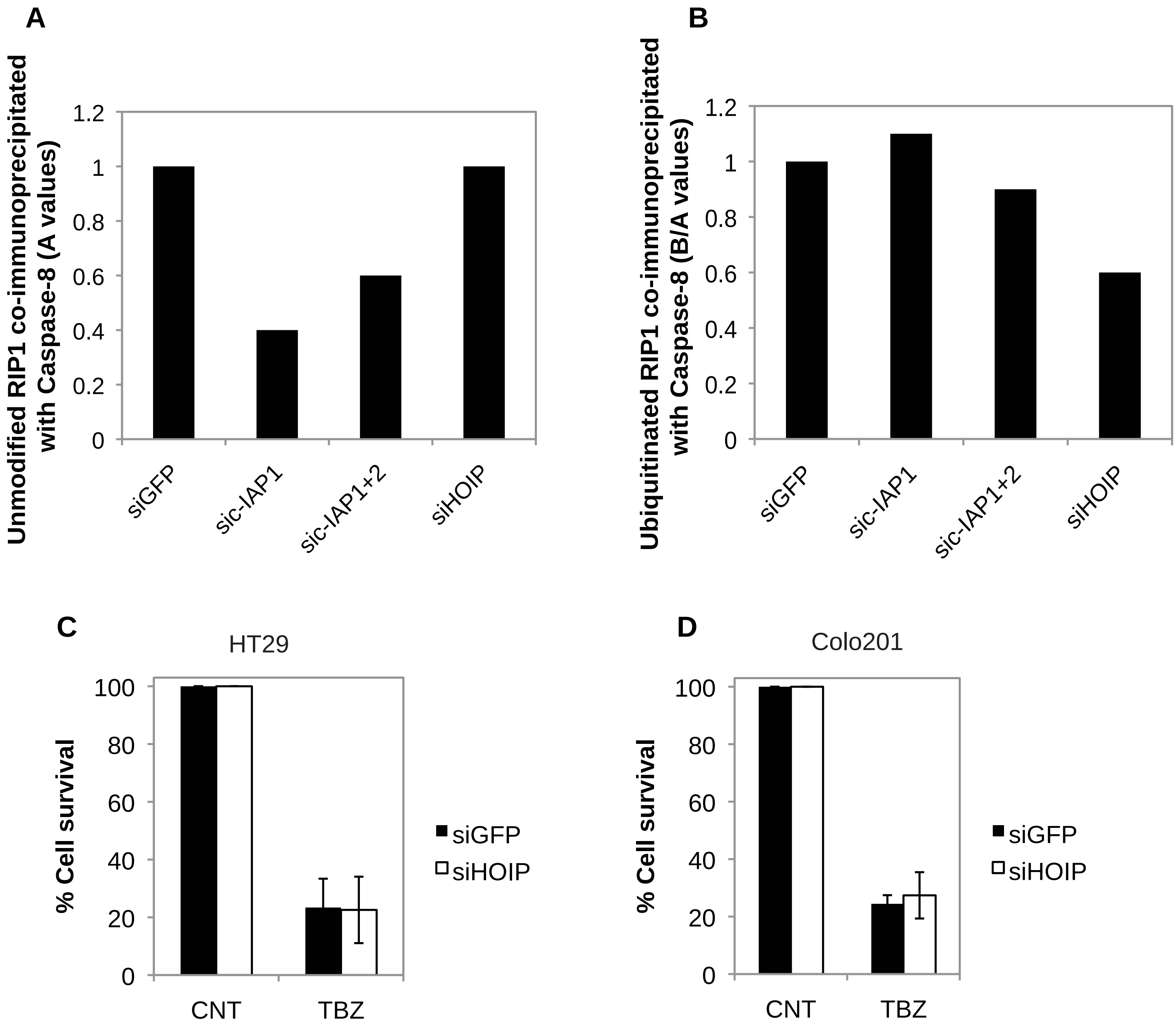

Figure S8, de Almagro et al.

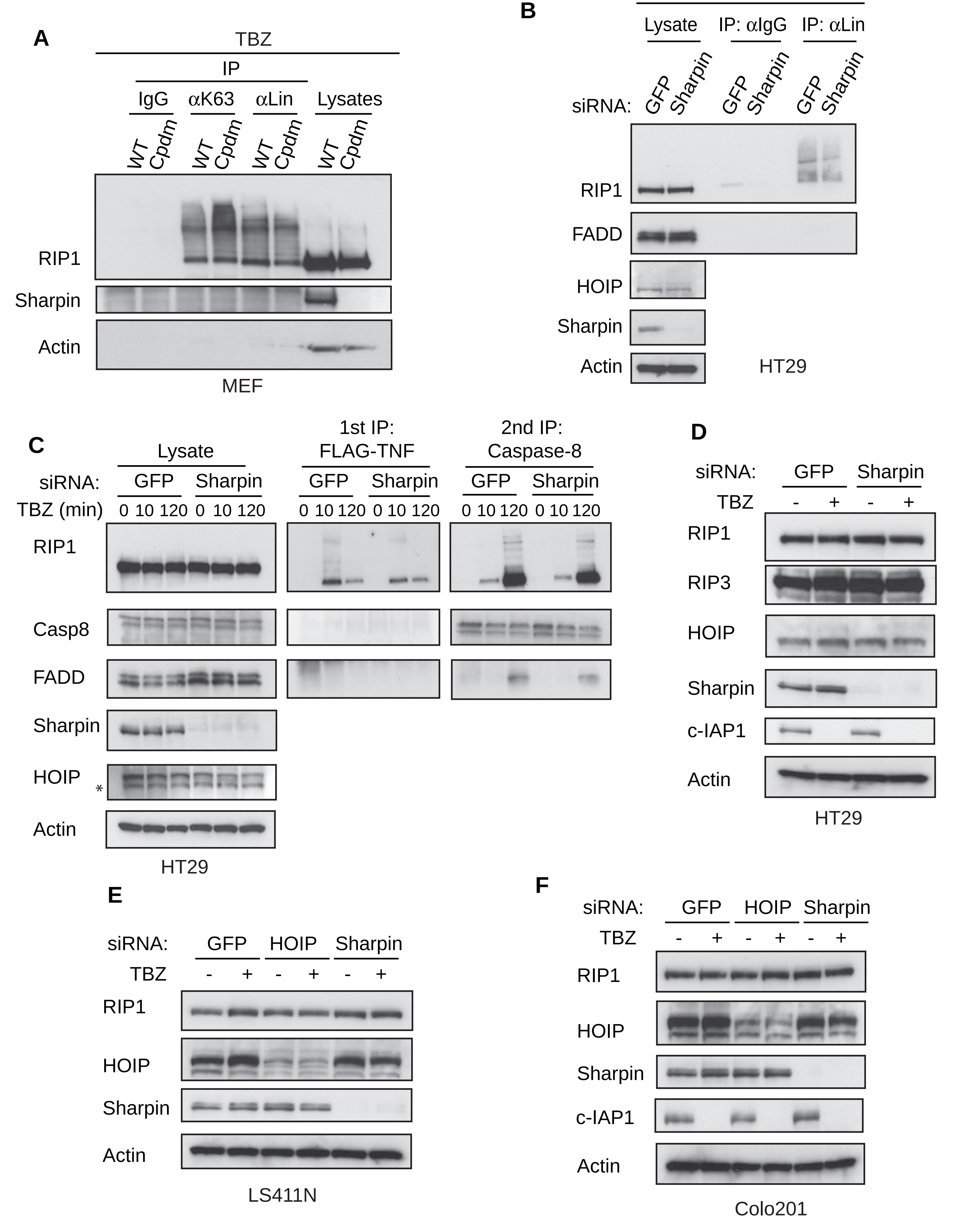

Supplement: Supplementary Information [file cddis2015158x1.pdf]
